# Magnitude and Impact of Hallucinations in Tabular Synthetic Health Data on Prognostic Machine Learning Models: Validation Study

**DOI:** 10.2196/77893

**Published:** 2025-08-18

**Authors:** Lisa Pilgram, Samer El Kababji, Dan Liu, Khaled El Emam

**Affiliations:** 1 School of Epidemiology and Public Health Faculty of Medicine University of Ottawa Ottawa, ON Canada; 2 CHEO Research Institute Children's Hospital of Eastern Ontario Ottawa, ON Canada; 3 Department of Nephrology and Medical Intensive Care Charité - Universitaetsmedizin Berlin Berlin Germany

**Keywords:** synthetic data, data utility, hallucinations, generative models, artificial intelligence, AI

## Abstract

**Background:**

Generative artificial intelligence (AI) for tabular synthetic data generation (SDG) has significant potential to accelerate health care research and innovation. A critical limitation of generative AI, however, is hallucinations. Although this has been commonly observed in text-generating models, it may also occur in tabular SDG.

**Objective:**

This study aims to investigate the magnitude of hallucinations in tabular synthetic data, whether their frequency increases with training data complexity, and the extent to which they impact the utility of synthetic data for downstream prognostic machine learning (ML) modeling tasks.

**Methods:**

On the basis of 12 large and high-dimensional real-world health care datasets, 6354 training datasets of different complexity were created by varying the subset of variables included in each dataset. Synthetic data were generated using 7 different SDG models. Hallucinations were defined as synthetic records that did not exist in the population, and the hallucination rate (HR) was the proportion of hallucinations in a synthetic dataset. Classification was the downstream prognostic modeling task, conducted via an ML approach (light gradient boosted machine) and an artificial neural network (multilayer perceptron). Mixed-effects models were fitted to examine the relationship between training data complexity and the HR and the HR and the predictive performance of AI and ML models when trained on the synthetic data.

**Results:**

The HR ranged from 0.3% to 100% (median 99.1%, IQR 98.5%-100.0%) and increased with training data complexity. However, in most SDG models, the HR did not affect AI and ML prognostic model performance. In the SDG models in which a significant association was detected, the estimated effect was very small, with a maximum decrease in the area under the receiver operating characteristic curve of –0.0002 (95% CI –0.0003 to –0.0002, *P*<.001) in light gradient boosting machine and –0.0001 (95% CI –0.0002 to –0.0001, *P*=.002) in multilayer perceptron.

**Conclusions:**

These findings suggest that while hallucinations may be very common in synthetic tabular health data, they do not necessarily impair its utility for prognostic modeling.

## Introduction

Generative models are a class of artificial intelligence (AI) and machine learning (ML) models that create new data from the input data they were trained on. During the training process, generative models learn the underlying joint probability distribution of the training data and sample output data from that distribution.

### Hallucinations in Generative Image and Text Modeling

The term “hallucination” in generative modeling first appeared in the context of creating high-resolution images from low-resolution input [1]. It described the ability of a model to generate output that exceeded the information learned from its input. This was considered a positive feature as face recognition or verification applications required high-resolution images; yet, often only low-resolution images were available. Models that generated such hallucinations were able to output high-resolution face images based on a lower-quality input and were built upon convolutional neural networks [2] or generative adversarial networks [3-11].

With the rise of large language models (LLMs), such as generative pretrained transformers, the term “hallucination” became more popular and took on the meaning that we currently use. It describes a specific form of generated output that can be seen as implausible, inconsistent, or nonexistent. Ji et al [12] define it as “generated content that is nonsensical or unfaithful to the provided source content.” This means hallucinations distinguish themselves from other types of output by a certain degree of unexpectedness and a higher deviation from training data. Today, 2 different notions of hallucinations are commonly used. The first one captures violations of the concept of *factuality* where the real world is used as the benchmark, while the second one is based on *faithfulness,* which describes consistency and truthfulness to the training data [12].

Hallucinations in the context of LLMs are largely seen as problematic. Multiple authors warn of overreliance on LLMs, particularly due to potential hallucinations that may be misleading [13-15]. In evaluation studies across various sectors, generic LLMs were shown to produce hallucinations [16-18]. For example, nontrivial deviations from the real world have been detected in generated scientific reports [19], and LLMs have been found to have limited ability to provide genuine references [20-22].

### The Challenge With Hallucinations in Health Care

Hallucinations are particularly harmful in fields such as medicine where there is little room for error and decisions can have severe consequences [14,23-25]. The National Academies of Sciences, Engineering, and Medicine consequently lists hallucinations as one of the major risks of generative AI in the health care sector, alongside concerns such as privacy, bias, output limitations, and algorithmic brittleness [14]. Medical hallucinations in the context of LLMs have been broadly defined as “incorrect or misleading medical information that could adversely affect clinical decision making and patient outcomes” [25].

This definition encompasses the notion of *factuality* as it evaluates the generated content against the real world. In addition, it extends beyond *factuality* by including any medical information that is misleading, such as biased conclusions or reasoning errors, and explicitly considering the potential harm that may result from such hallucinations. This broader definition shows that in the health care sector, LLM-generated hallucinations are viewed primarily through the lens of potential harmful consequences. Such consequences can be related to patient safety but also include the erosion of trust in AI and ML systems, increased workload or workflow disruptions in clinical settings, and unresolved ethical and legal questions about accountability [14,25].

### Hallucinations in Generative Tabular Modeling

Synthetic data generation (SDG) represents another form of generative modeling where synthetic tabular data are created by a model. Although SDG can be based on distributions known a priori and informed by background knowledge, published summary statistics, or established risk calculators [26-30], our focus here is on synthetic data generated based on a real dataset that is used to train a generative model, which outputs a fully synthetic tabular dataset.

Most research in tabular SDG focuses on improving and evaluating SDG models in terms of utility, privacy, and fairness [31,32]. The goal is to mimic the statistical properties of real data while maintaining low disclosure risks and avoiding bias in the generated synthetic data to ultimately ensure that the synthetic data perform well in downstream tasks. However, the concept of hallucinations has not been precisely defined or evaluated in the context of tabular SDG.

### Objectives

This study aimed to evaluate (1) the extent to which generated synthetic health data contain hallucinations, which has not been previously studied; (2) the impact of dataset complexity on the occurrence of hallucinated records, the hypothesis being that datasets with higher complexity will have a higher rate of hallucinations; and (3) the association between the rate of hallucinations and the performance of prognostic AI and ML models, the hypothesis being that the greater the rate of hallucinations, the less effective the prognostic models would be.

## Methods

### Definition of Hallucinations in Tabular Synthetic Data

**Figure 1:**
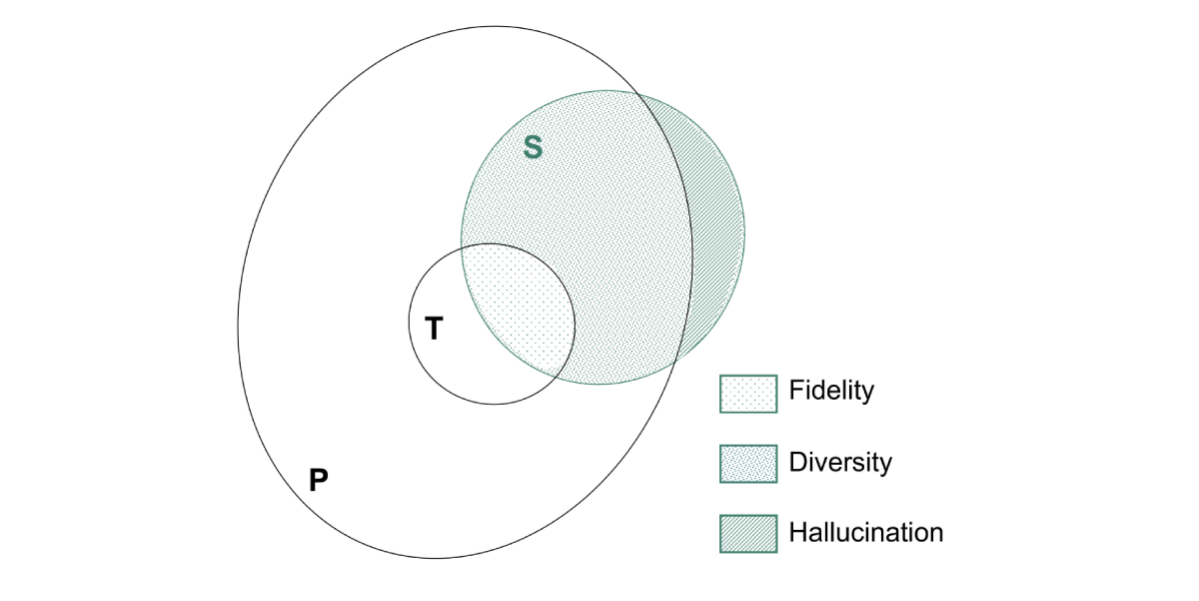
Hallucinations in synthetic data. The green circle represents the synthetic data (S). Within S, high-fidelity records are synthetic records that are similar to the training data (dotted portion); diverse records are the ones that are not similar to the training data (T) but to the population (P; dense dotted portion); hallucinated records are those that cannot be considered as being representative of the training or the population data (striped portion).

Utility in synthetic data has been typically defined in terms of fidelity and downstream utility. Fidelity means that the synthetic data are similar to the training data, and metrics can be used to indicate how close the records are [33-35]. For example, the Hellinger distance measures similarity in multivariate distributions; the cluster metric compares the clustering structure [34]. The training dataset serves as a basis for comparison, and high-fidelity synthetic data are data that resemble the training data very well. This is similar to the aforementioned concept of *faithfulness*. A violation of fidelity can be seen as diversity (Figure 1). Diverse records are those that are not similar to the training data but are still quite similar to the population from which the training data were drawn. In SDG, the goal is typically not to have complete *faithfulness* to the training data, as this could expose individuals’ personal information. Instead, diverse records that maintain the statistical properties of the population can be privacy preserving while supporting, for example, a prognostic model to generalize better and perform reasonably well on unseen data from the same population.

Hallucinations in tabular synthetic data can be defined as synthetic records that are nonexistent in the population (Figure 1). This can be because they are implausible (eg, a female individual with prostate cancer) or are plausible but just do not exist in the population (eg, there is no male individual in a specific population of patients with breast cancer). It thereby incorporates the concept of *factuality* rather than *faithfulness* as the evaluation is performed with reference to the population and not the training dataset [36].

It has been argued that hallucinations represent the low-likelihood outputs of a model [37]. Consequently, as for any generative model, we can assume that SDG leads to hallucinated records. However, it is unknown to what extent this happens in tabular SDG. In addition, one can reasonably argue that training a prognostic model on datasets with hallucinated records may degrade the performance of the model on unseen (ie, holdout) data, as the model would learn patterns that are not, and cannot be, in unseen data from the same population. Therefore, in addition to hallucinations eroding trust in synthetic data, they may have the practical consequence of reducing the performance of at least prognostic analytic workloads with the synthetic data.

### Study Workflow

The overall workflow of this study included five major steps:

1. Creation of population variants with varying complexity from 12 real-world health care populations
2. Sampling a training dataset from each population variant to train 7 different SDG models, spanning from more traditional statistical to deep learning models
3. Generating 10 synthetic datasets from each trained SDG model and identification of hallucinated records in each of the synthetic datasets
4. Assessing the downstream predictive modeling performance in each of the synthetic and training datasets via light gradient boosted decision trees (LGBM) and multilayer perceptron (MLP)
5. Estimating the effect of complexity on hallucinations as well as the effect of hallucinations on downstream modeling

**Figure 2:**
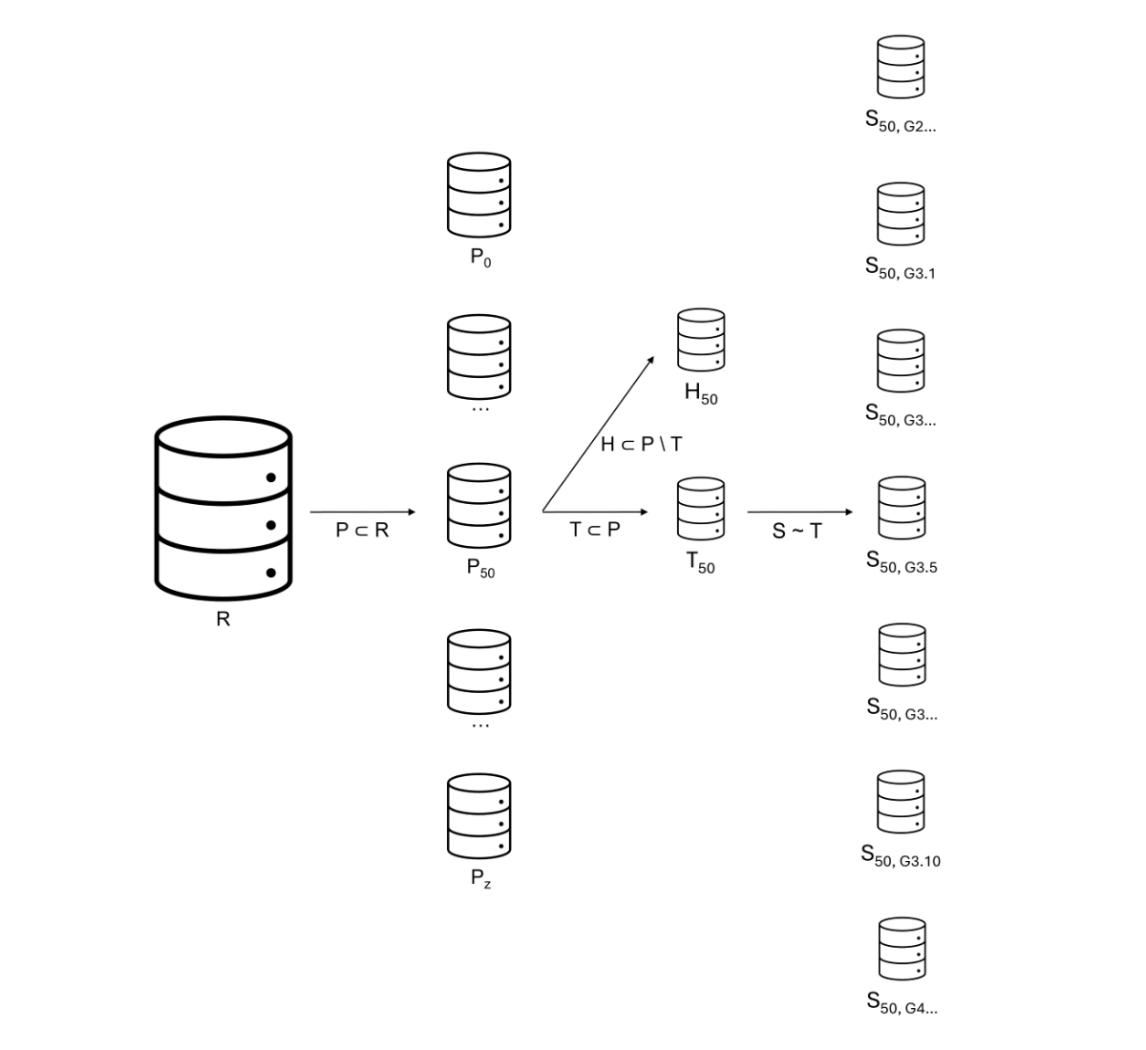
Creation of population (P) variants and synthetic data generation (SDG). For each real-world health care reference population (R), a core population was defined as P0 and included the core variables as defined by the downstream modeling task of R. By varying numbers and combinations of adjunct variables, z additional population variants with different levels of complexity were created (P1-Pz) so that each variant was a subset of R (P⊂R). The number of records remained the same. From these population variants, the SDG training dataset (T) was taken (subset T⊂P). The holdout dataset (H) was a disjoint subset from the same population variant, explicitly excluding all records used in the training dataset (H⊂P \ T). Across all variants, the same subset of records was selected as training and holdout datasets, respectively. Ten synthetic datasets (eg, G3.1-G3.10) were generated per SDG model (G1-G7). ⊂: proper subset (subset of randomly drawn or selected records); \: complement; ~: SDG; G: generator (SDG model).

The creation of population variants from real-world health care populations and subsequent SDG (steps 1 and 2) is demonstrated in Figure 2 and can be summarized as mentioned subsequently. For each real-world population, diverse population variants with the same records but varying numbers and combinations of variables were created to capture a large space of dataset complexity. A random sample of 10,000 records was then defined as a training dataset and a disjoint random sample of 10,000 records as a holdout dataset. From each population variant, the same training and holdout sample was taken to train the 7 SDG models and generate 10 synthetic datasets each to account for the stochasticity of the generative process.

All steps were conducted in parallel on containerized execution environment within the hospital high-performance computing infrastructure with a total of 13 graphics processing units (NVIDIA RTX A6000, each with 48 GB of memory) and 256 central processing unit cores (1 TB of available memory). Runtime varied depending on the complexity of the population variant and the SDG model, with steps 2 and 4 being the most computationally demanding steps in the overall workflow. For 1 population variant, the runtime of step 2 (ie, SDG via 7 SDG models) varied between 180 seconds and 3780 seconds (depending on the complexity of the population variant) and the runtime of step 4 (ie, downstream model training) between 46 seconds and 104 seconds for LGBM and between 62 seconds and 139 seconds for MLP (depending on the downstream task). The runtime of step 5 (ie, effect estimation across all population variants and SDG models) took approximately 1800 seconds in total.

### Creation of Population Variants

For this study, large datasets were needed to simulate a reference population. We used the real-world datasets listed in Table 1. These datasets cover a wide range of typical characteristics (eg, class imbalance, missing values, and noisy variables) that are encountered when working with real-world health data [38,39]. Furthermore, the datasets cover multiple domains, including hospital discharge, adverse events, public health, health surveys, and population registries.

In this study, we use the term *reference population* to refer to the real-world dataset with its full set of records and variables. We hypothesized that the complexity of a dataset would contribute to the occurrence of hallucinations. To capture various complexities for one reference population, we derived *population variants* from it by varying its dimensionality. These population variants were built by adding *adjunct* variables to a *core* set of variables, and we refer to the dataset with the *core* variables as the *core* population. This means that population variants shared the same (entire) set of records but included different subsets of variables. The general term *population* refers to their provenance (ie, the reference population) and is used as a label for grouping rather than to describe any particular dataset. The *core* variables were determined by the downstream modeling task and are specific to the population. Details on the datasets, their downstream modeling tasks, and the core variables of each are provided in Multimedia Appendix 1 [23-28,40-99].

Depending on the original dimensionality of the reference population, the selection of the combinations of *adjunct* variables would result in a large combinatorial space, as discussed subsequently.

**Table 1:** Characteristics of real-world populations^a^.

Population	Brief description	Core^b^ variables, n	Pool size^c^, n	Variants^d^, n	Reference Population size, n
BORN^e^	Birth registry in the province of Ontario, Canada, with information about pregnancy and birth	20	101	700	968,435
California hospital discharges 2008 (California)	Discharge dataset from hospitals in California, United States, from 2007	15	387	601	4,017,998
CCHS^f^	Canadian population survey with health information	13	121	723	904,813
Canadian COVID-19 (COVID-19)	Canadian COVID-19 dataset	6	5	32	1,384,881
FAERS^g^	Dataset of adverse events submitted to the FDA^h^, United States	9	27	614	881,204
Florida hospital discharges 2007 (Florida)	Discharge dataset from hospitals in Florida, United States, from 2007	10	293	601	2,563,370
MIMIC-III^i^	Data from intensive care unit admissions of the Beth Israel Deaconess Medical Center, United States	13	4	16	30,662
New York hospital discharges 2007 (New York)	Discharge dataset from hospitals in New York, United States, from 2007	13	317	601	2,608,615
Nexoid COVID-19 survival calculator data (Nexoid)	Web-based survey data concerning COVID-19 provided by a company in London, United Kingdom	19	36	622	968,408
Texas inpatient public use data file (Texas)	Discharge dataset from hospitals in Texas, United States	10	65	642	745,999
Washington state hospital discharges 2007 (Washington)	Discharge dataset from hospitals in Washington, United States, from 2007	8	349	601	644,902
Washington state hospital discharges 2008 (Washington 2008)	Discharge dataset from hospitals in Washington, United States, from 2008	17	407	601	652,344

^a^The reference populations were transformed to be based on individual-level (not event-level) observations. For the Better Outcomes Registry & Network population, the individual was the newborn. The exception was the US Food and Drug Administration Adverse Event Reporting System, which could not be transformed due to the absence of a unique identifier; however, given that adverse events are rare in general, it can be expected that there is a very low number of duplicate individuals.

^b^Core means the number of variables defined for their downstream task.

^c^Pool size is the total number of potential *adjunct* variables.

^d^Variants are subsets derived from the reference population by reducing it to the *core* variables and adding varying *adjunct* variables.

^e^BORN: Better Outcomes Registry & Network.

^f^CCHS: Canadian Community Health Survey.

^g^FAERS: US Food and Drug Administration Adverse Event Reporting System.

^h^FDA: US Food and Drug Administration.MIMIC-III: Medical Information Mart for Intensive Care III.

^i^MIMIC-III: Medical Information Mart for Intensive Care III.

We define *v*_0_ as the number of variables in the *core* dataset, so those are the ones that are required for a predefined downstream modeling task. *v* is the number of *adjunct* variables that are in the dataset but not required for the downstream modeling task. Then, the dimensionality of a dataset is defined by *v*_0_+*v*. The 12 reference populations had varying dimensionalities, so that the maximum number of potential *adjunct* variables varied. This is referred to as pool size *m*. The larger the pool size, the higher the total number of potential combinations. For example, if we want to create a dataset with 2 *adjunct* variables (ie, *v=*2) from a dataset that has 100 potential *adjunct* variables (ie, *m*=100), we have 
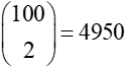
 distinct options to create a population variant by adding 2 *adjunct* variables to the *core* variables. If we considered all potential combinations for any given number of *adjunct* variables, the space of population variants would grow up to 1.267651×10^30^ distinct population variants in this example.

Therefore, to reduce the computational burden, we adopted a random weighted sampling scheme and analyzed, in total, 6354 variants derived from 12 health care reference populations. The sampling process is described in more detail in Multimedia Appendix 1.

### Measuring Complexity

While various complexity metrics for datasets have been described, many of them are specific to a downstream task, such as binary classification tasks [100,101]. Such metrics measure, for example, the discriminative power of each variable with respect to an outcome variable. As highlighted in the study by Cano [100], complexity metrics that include multiple different structural but also distributional characteristics can become challenging to interpret because very different datasets yield similar complexity values.

Sparsity measures that incorporate both the dimensionality and the size of the dataset focus on the structural characteristics of a dataset and offer a more straightforward interpretation [101]. In this study, all training datasets had the same number of records, making size-related information constant. At the same time, dimensionality alone seemed insufficient to comprehensively capture structural complexity.

Therefore, we considered cardinality, in addition to dimensionality, to obtain a more comprehensive but interpretable proxy for data complexity. The detailed definition, including the mathematical equation, can be found in Multimedia Appendix 1.

**Figure 3:**
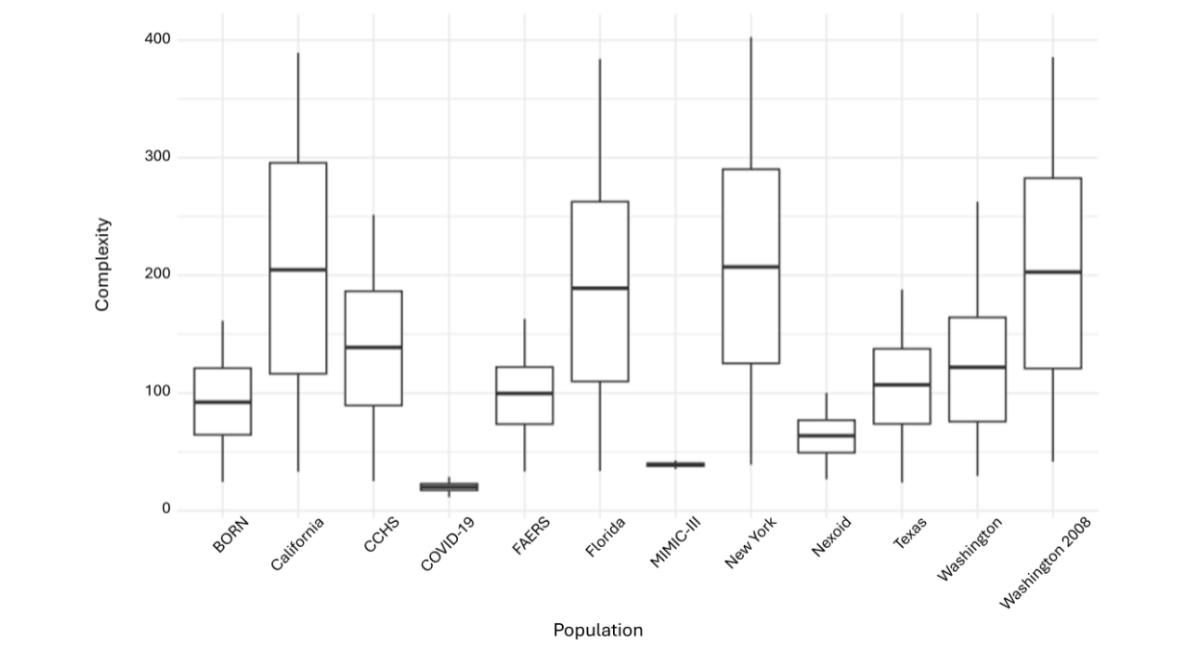
Complexity of population variants. Variants were created from the reference populations, as described, and complexity across all variants was calculated. The boxplots show the median as the central horizontal line, the lower and upper hinges represent the first and third quartiles (ie, IQR), and the whiskers represent the largest values within 1.5 times IQR from the quartiles. BORN: Better Outcomes Registry & Network; CCHS: Canadian Community Health Survey; FAERS: US Food and Drug Administration Adverse Event Reporting System; MIMIC-III: Medical Information Mart for Intensive Care III.

The population variants created in this study covered a large range of complexities, as depicted in Figure 3.

Figure 3 shows that only a few variants were of low complexity because *adjunct* variables often included high-cardinality variables (eg, diagnosis or medication), thereby increasing dataset complexity.

### SDG Models

In total, 7 different types of SDG models were considered when quantifying and analyzing hallucinations in SDG. In combination with the 6354 population variants, this gives 44,478 trained SDG models, each of which generated 10 synthetic datasets.

The 7 SDG models were sequential decision trees (STs) [102], Bayesian networks [103], conditional generative adversarial networks [104], variational autoencoders (tabular variational autoencoder and robust tabular variational autoencoder) [104], adversarial random forests [105], and normalizing flows [106]. The details on each of the SDG models are provided in Multimedia Appendix 1.

### Identification of Hallucinations

To assess hallucinations, we focused on the concept of *factfulness* in tabular SDG. Another concept is *faithfulness*. The difference between these concepts is the underlying ground truth. For instance, we will consider an abstractive summarization task, where a section from a travel guide about Canada should be summarized by an LLM. This section does not contain the explicit information that Ottawa is the capital of Canada but lists the biggest cities of Canada. Then, if the output states that Montreal is the capital of Canada, this can be classified as a hallucination in terms of *faithfulness* because the input data had no such information. It would also be considered a hallucination in terms of *factfulness* as it is not aligned with the ground truth. If the LLM’s output is that Ottawa is the capital of Canada based on the same input, this would also be classified as a hallucination in terms of *faithfulness* but not in terms of *factfulness*. This is because *faithfulness* is evaluated based on input data adherence, while *factfulness* requires an external ground truth, making its assessment more challenging [12].

In this study, we focus on *factfulness* because it provides a more meaningful interpretation in tabular synthetic data where some degree of diversity from the training data (so a violation of *faithfulness*) is both expected and desirable [107]. *Factful* synthetic records, in contrast, are records that appear in the population variant where the training data are sampled from but may or may not be in the training data. In this study, we then define hallucinations in terms of *factfulness* as synthetic records that are nonexistent in the population variant from which the training data were sampled. This includes records that may be statistically consistent with the distribution of the training data but which nonetheless never appeared in the actual population variant.

This definition has a clearer interpretation than alternative definitions that rely on semantic or probabilistic similarity and require the specification of thresholds. Such thresholds are difficult to define, particularly in our context where precedents are lacking, and have a nontrivial impact on interpretability. Our definition should also, in principle, be more sensitive than these alternative approaches. However, it is important to note that alternative definitions may lead to different conclusions, as discussed in the Strengths and Limitations section.

To operationalize our definition, we singled out synthetic records that were nonexistent in the corresponding population variant by matching records between the synthetic and population variant and isolating those that were uniquely present in the synthetic data. The set of hallucinated records (HA) is then the difference between the synthetic data (S) and the population variant (P), calculated as follows:

*HA* = *S \ P*

More precisely, we applied row-wise antijoin between the synthetic data and the population variant (implemented via the *dplyr* package [108] in R software [R Foundation for Statistical Computing]), which returned those records from the synthetic data that did not have an exact match in the corresponding population variant. This definition is functionally equivalent to a record-level Hamming distance of more than 0 from the synthetic to the closest real record. However, rather than calculating row-wise distances, we used exact match comparison, which is computationally simpler and more efficient. Treatment of missing values and numerical variables is detailed in Multimedia Appendix 1.

The parameter of interest for this study was the hallucination rate (HR) in a synthetic dataset, defined as follows:



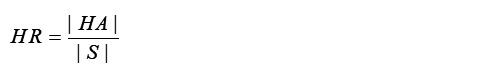



whereby |*HA*| was the number of hallucinated records and |*S*| was the size of the synthetic dataset (ie, 10,000 records). The HR was averaged across the 10 synthetic datasets per trained SDG model.

### Downstream Task Performance

Downstream utility was defined as prognostic AI and ML modeling performance and was assessed by train-synthetic-test-real (TSTR) utility [109]. TSTR utility is when a prediction model is trained on the synthetic data and then tested on unseen real records (ie, holdout dataset) to see if it can make correct predictions [109]. Accurately modeling a population is the very aim of research, and TSTR is a very meaningful metric to evaluate the utility of a synthetic dataset.

The holdout dataset was composed of 10,000 random records, disjoint from the training dataset and fixed across all population variants for each real-world health care population to allow for comparability across the variants and between synthetic and real data. This corresponds to a 50:50 split for the training and holdout datasets. Importantly, to avoid any information leakage, the holdout dataset was not only independent from prognostic model training but also from SDG model training. To investigate the sensitivity to the single 50:50 split, the downstream performance of the real data was calculated over 10 additional splits. Results are detailed in Multimedia Appendix 1 and show that there was little variation across the splits.

All reference populations came with a predefined binary classification task involving the *core* variables. A binary classification model was built using LGBM, which is a commonly applied ML prediction model [110,111]. Tree-based models are the most common type of ML prognostic methods used in clinical research [112]; they perform better than linear models, such as logistic regression [113-117], and have also been found to perform better than deep learning models on tabular datasets [118,119]. In addition, we trained an MLP to account for contemporary neural network classification approaches. Model performance was assessed as the area under the receiver operating characteristic curve (AUROC) [120] and averaged across the 10 synthetic datasets per trained SDG model.

In LGBM, hyperparameters were chosen based on AUROC in 5-fold cross-validation during model training [121]. Details with respect to the implementation and hyperparameters to select from are described in Multimedia Appendix 1.

In MLP, we built a sequential classification model with an input layer with 16 nodes, a dropout layer, a second hidden layer with 16 nodes, and an output layer with 1 node and a sigmoid activation function for binary classification. Extensive hyperparameter tuning was not conducted, as exploratory results already demonstrated that this setup yields comparable results to LGBM. We focused instead on avoiding overfitting [40]. Details with respect to the implementation and overfitting avoidance are described in Multimedia Appendix 1.

In addition, we measured performance when using the real (training) dataset for prognostic modeling (ie, train real test real). This gave us the performance that would be possible when using real data instead of synthetic data and served as a reference point.

In total, 451,134 LGBM models and 451,134 MLP models were trained.

### Effect Estimation

We analyzed the association between data complexity and hallucinations (ie, HR) as well as hallucinations and downstream utility (ie, TSTR). We estimated the effect for each SDG model separately.

Initial modeling results suggested that there was an unobserved (ie, random) effect beyond complexity contributing to HR and an effect beyond HR contributing to TSTR. This can be explained by the unique distribution, unique *core* variables, and the specific downstream tasks of each of the 12 populations.

Random effects can be captured by mixed-effects models. Such models assess a fixed component while accounting for a random component. In this study, the random component was the provenance of the population variant, which was the 12 health care populations. We estimated the fixed effect of complexity on the outcome HR as well as the fixed effect of HR on the outcome TSTR. When estimating the effect of HR on TSTR, we only considered those populations with sufficient spread in the HR across all population variants, more precisely, where the difference between the 10th and 90th percentiles of HR was at least 0.25. Details on the models and their implementation are provided in Multimedia Appendix 1.

The level of significance was chosen to be .05. The odds ratio (OR) with respective 95% CI is reported as effect size for generalized linear mixed-effects models and the coefficient (or effect estimate) with respective 95% CI for linear mixed-effects models. We evaluated model fit using marginal and conditional *R*^2^ values. These quantify the variance explained by the fixed effects alone and by both fixed and random effects [122].

Given the large scale of our experiments, an important question is whether such a large number of population variants is needed to estimate the effects as described earlier. These secondary (or sensitivity) analyses confirmed the robustness of effect estimation but, more importantly, can inform potential design adjustments in terms of scale in future methodological research. They were conducted by randomly selecting 50% and 25% of the population variants for each reference population. More precisely, from the entire set of population variants per real-world reference population, we chose a random subset of 50% and 25% and used the mixed-effects models as described earlier to estimate the fixed effects of complexity on the outcome HR as well as the fixed effects of HR on the outcome TSTR. The detailed results are presented in Multimedia Appendix 1.

### Ethical Considerations

This project has been approved by the Children’s Hospital of Eastern Ontario Research Institute Research Ethics Board (REB) protocol (24/103X).

The Children’s Hospital of Eastern Ontario REB operates in compliance with, and is constituted in accordance with, the requirements of the Tri-Council Policy Statement: Ethical Conduct of Research Involving Humans [123]; the International Conference on Harmonization Good Clinical Practice Consolidated Guideline [124]; part C, division 5 of the Food and Drug Regulations [125]; part 4 of the Natural Health Products Regulations [126]; and part 3 of the Medical Devices Regulations [127] and the provisions of the Ontario Personal Health Information Protection Act 2004 and its applicable regulations [128].

This research involved the secondary use of deidentified health care datasets originally collected for purposes other than this study. This made the potential of disclosure risks the primary ethical consideration of this study. However, all datasets were deidentified at the source by the respective data custodians and were assessed as low risk. All analyses were conducted within a secure server environment with access restricted to authorized researchers of this study. These researchers have completed institutional privacy and security training, including instruction on the appropriate handling of personal health information, and, where required by data custodians, researchers also agreed to specific terms of use and completed additional ethics or data governance training. In accordance with the Tri-Council Policy Statement: Ethical Conduct of Research Involving Humans [123], individual reconsent was waived by the REB given that secondary use of deidentified data in this study posed minimal risk.

## Results

### Hallucinations During SDG

We analyzed the HR when generating tabular health data. The minimum HR was 0.3%, and the maximum HR was 100%. We found that the median (99.1%, IQR 98.5%-100.0%) HR across all synthetic datasets was very high. This finding remained consistent when applying an alternative operational implementation of the HR (Multimedia Appendix 1).

Complexity had a fixed effect on the HR via generalized linear mixed-effects modeling with the population as a random effect. More precisely, for each SDG model, there was a significant positive association between the complexity of the (training) data and the HR. The OR ranged from 1.07 (95% CI 1.03-1.11) in ST to 1.16 (95% CI 1.11-1.22) in normalizing flows. As shown in Table 2, the contribution of the HR as a fixed effect to the explained variance varied across the SDG models, and the random effect was consistently a large part of the total explained variance.

**Figure 4:**
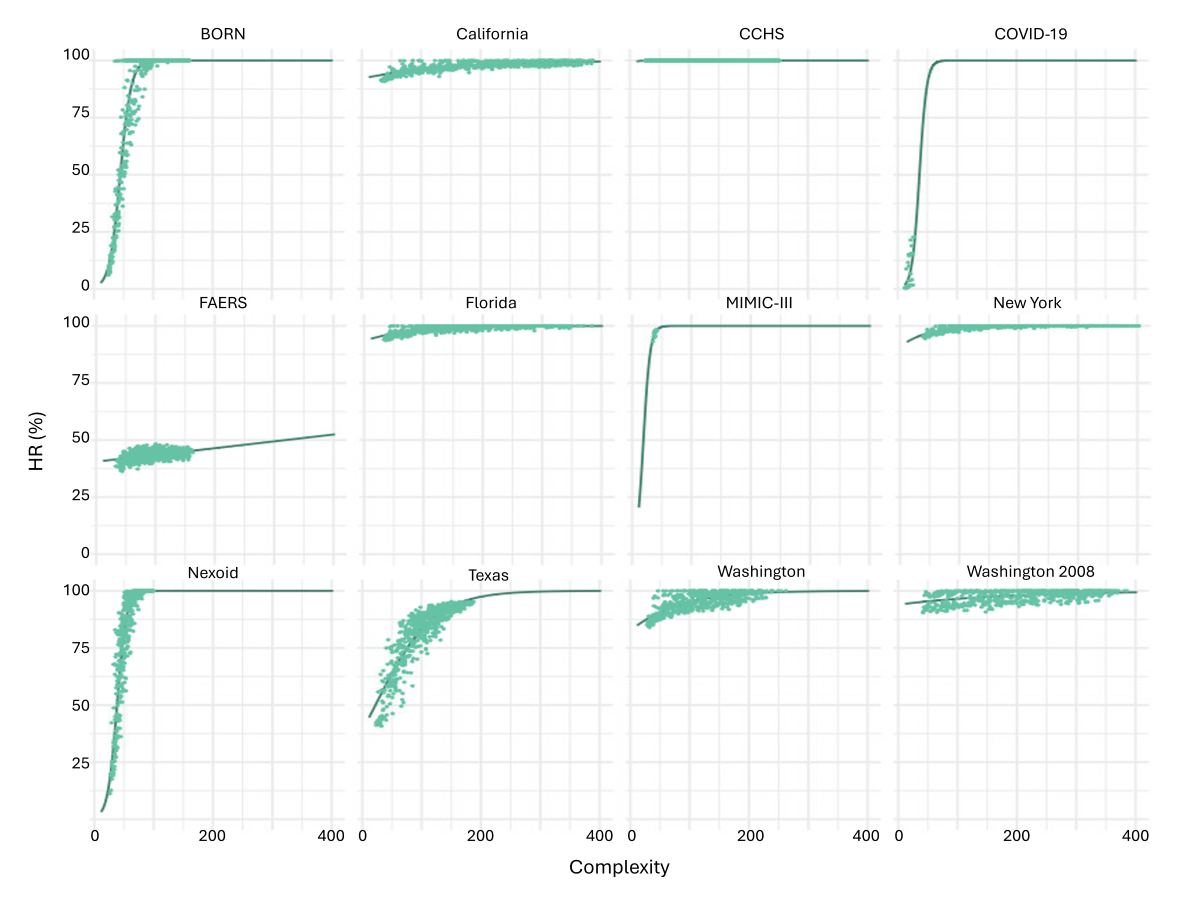
Mixed-effects model with the population as a random effect, complexity as a fixed effect, and hallucination rate (HR) as an outcome for the synthetic data generation (SDG) model sequential decision trees (STs). HR in synthetic datasets was determined as described and averaged across the 10 synthetic datasets per trained SDG model. Complexity for a dataset was calculated as the log sum of its variables’ cardinalities. The lines are the predicted HR by the mixed-effects model, while the points are the observed HR. BORN: Better Outcomes Registry & Network; CCHS: Canadian Community Health Survey; FAERS: US Food and Drug Administration Adverse Event Reporting System; MIMIC-III: Medical Information Mart for Intensive Care III.

In Figure 4, the behavior of the SDG model with the lowest HR (ie, ST) is illustrated across the different populations. Notably, the effect can add up considerably with increasing complexity. As shown in Table 2, this effect is similar for the other SDG models.

The fixed effect of complexity on the HR was also modeled with fewer population variants (ie, a 50% and 25% subset) as a sensitivity analysis to the sample size. The effect sizes of these sensitivity analyses were very similar to the main analysis, confirming the robustness of our results in a smaller-scale evaluation setup (Multimedia Appendix 1).

**Table 2:** Modeling the effect of complexity on the hallucination rate^a^.

SDG^b^ model	Fixed effect complexity, OR^c^ (95% CI)	*P* value	*R*^2^ (fixed effect)	*R*^2^ (overall)
ST^d^	1.07 (1.03-1.11)	<.001	0.26	0.99
BN^e^	1.03 (1.01-1.05)	.001	0.16	0.99
ARF^f^	1.07 (1.03-1.12)	<.001	0.29	0.99
CTGAN^g^	1.11 (1.08-1.14)	<.001	0.57	0.99
TVAE^h^	1.11 (1.07-1.15)	<.001	0.47	0.99
RTVAE^i^	1.16 (1.10-1.23)	<.001	0.45	0.99
NFlow^j^	1.16 (1.11-1.22)	<.001	0.54	0.99

^a^Generalized linear mixed-effect models were fitted for each synthetic data generation model separately, with the following number of observations: 6354 for sequential decision trees; 6354 for Bayesian network; 6354 for adversarial random forests; 6354 for conditional generative adversarial network; 6354 for tabular variational autoencoder; 6353 for robust tabular variational autoencoder; and 6328 for normalizing flow. The population was considered as a random effect, complexity as a fixed effect, and the HR as an outcome. The odds ratios for hallucinations are indicated. We provide the variance explained (ie, *R*^2^) by the fixed effect only and by both fixed and marginal effects together (ie, *R*^2^ overall) for all models.

^b^SDG: synthetic data generation.

^c^OR: odds ratio.

^d^ST: sequential decision tree.

^e^BN: Bayesian network.

^f^ARF: adversarial random forest.

^g^CTGAN: conditional generative adversarial network.

^h^TVAE: tabular variational autoencoder.

^i^RTVAE: robust tabular variational autoencoder.

^j^NFlow: normalizing flow.

### Downstream Prognostic AI and ML Modeling

Once the occurrence of hallucinations in tabular synthetic health care data was confirmed, we analyzed the effect of HR on downstream utility. The downstream task was prognostic AI and ML modeling, and performance was measured by AUROC when LGBM and MLP models were trained on synthetic and tested on real data (ie, TSTR).

In general, the median deviation of the AI and ML performance derived from the synthetic data (ie, TSTR) from the one derived from the real data (ie, train real test real) was low across all health care populations (Table 3). Notably, in the Nexoid population, most prognostic MLP models trained on synthetic data outperformed the model trained on real data (refer to Table 3 and green vs dashed gray lines in Figure 4).

Train real test real was calculated across 10 additional training-holdout splits to investigate sensitivity to the stochasticity of the data partitioning. The variation was very small for LGBM across all populations and also for MLP, except in the US Food and Drug Administration Adverse Event Reporting System (Multimedia Appendix 1). This indicates that performance was generally robust and insensitive to the particular data split used.

**Table 3:** Downstream prognostic artificial intelligence and machine learning modeling performance^a^.

Population	LGBM^b^				MLP^c^		
	TRTR^d^, median (IQR)	TSTR^e^, median (IQR)	TRTR-TSTR, median (IQR)	TRTR, median (IQR)		TSTR, median (IQR)	TRTR-TSTR, median (IQR)
BORN^f^	0.923 (0.922 to 0.924)	0.906 (0.899 to 0.911)	0.016 (0.011 to 0.025)	0.896 (0.896 to 0.896)		0.864 (0.850 to 0.876)	0.032 (0.020 to 0.046)
California	0.810 (0.809 to 0.812)	0.666 (0.631 to 0.721)	0.144 (0.089 to 0.176)	0.854 (0.854 to 0.854)		0.824 (0.804 to 0.839)	0.030 (0.015 to 0.050)
CCHS^g^	0.708 (0.706 to 0.710)	0.664 (0.639 to 0.682)	0.043 (0.026 to 0.068)	0.698 (0.698 to 0.698)		0.694 (0.688 to 0.698)	0.004 (0.000 to 0.010)
COVID-19	0.957 (0.954 to 0.959)	0.771 (0.609 to 0.917)	0.187 (0.038 to 0.349)	0.931 (0.931 to 0.931)		0.740 (0.661 to 0.829)	0.192 (0.103 to 0.270)
FAERS^h^	0.663 (0.652 to 0.675)	0.557 (0.538 to 0.574)	0.105 (0.086 to 0.127)	0.928 (0.928 to 0.928)		0.818 (0.770 to 0.863)	0.110 (0.064 to 0.157)
Florida	0.750 (0.748 to 0.751)	0.622 (0.596 to 0.644)	0.127 (0.106 to 0.154)	0.837 (0.837 to 0.837)		0.811 (0.789 to 0.825)	0.026 (0.011 to 0.048)
MIMIC-III^i^	0.654 (0.653 to 0.658)	0.561 (0.547 to 0.571)	0.094 (0.080 to 0.108)	0.534 (0.534 to 0.534)		0.527 (0.522 to 0.533)	0.008 (0.002 to 0.013)
New York	0.806 (0.801 to 0.806)	0.651 (0.626 to 0.686)	0.153 (0.118 to 0.178)	0.859 (0.859 to 0.859)		0.832 (0.811 to 0.848)	0.027 (0.012 to 0.049)
Nexoid	0.730 (0.729 to 0.731)	0.676 (0.662 to 0.702)	0.054 (0.029 to 0.068)	0.681 (0.681 to 0.681)		0.683 (0.671 to 0.692)	–0.002 (–0.011 to 0.010)
Texas	0.810 (0.808 to 0.811)	0.747 (0.720 to 0.762)	0.062 (0.048 to 0.090)	0.813 (0.813 to 0.813)		0.788 (0.778 to 0.800)	0.025 (0.012 to 0.035)
Washington	0.784 (0.782 to 0.788)	0.650 (0.617 to 0.679)	0.135 (0.105 to 0.167)	0.870 (0.870 to 0.870)		0.844 (0.831 to 0.852)	0.026 (0.017 to 0.038)
Washington 2008	0.808 (0.806 to 0.810)	0.684 (0.649 to 0.709)	0.125 (0.100 to 0.160)	0.877 (0.877 to 0.877)		0.843 (0.827 to 0.857)	0.034 (0.019 to 0.050)

^a^The different downstream tasks achieved varying performance in the real data (train real test real). The deviation of the performance derived from the synthetic data (train synthetic test real) is indicated as TRTR-TSTR. Performance was measured as the area under the receiver operating characteristic curve. The train synthetic test real is summarized across all synthetic data generation models.

^b^LGBM: light gradient boosted decision tree.

^c^MLP: multilayer perceptron.

^d^TRTR: train real test real.

^e^TSTR: train synthetic test real.

^f^BORN: Better Outcomes Registry & Network.

^g^CCHS: Canadian Community Health Survey.

^h^FAERS: US Food and Drug Administration Adverse Event Reporting System.

^i^MIMIC-III: Medical Information Mart for Intensive Care III.

To detect a trend in HR on TSTR, we focused on those populations where the HR differed across the variants at least by 0.25 between the 10th and 90th percentiles. While TSTR was computed for all synthetic datasets, this filtering step reduced the subset used for effect modeling to 19.71% (8766/44,478 trained SDG models).

Among these, TSTR from LGBM was not affected by HR in most SDG models (6/7, 86%). Only the conditional generative adversarial network showed a significant decrease in prognostic LGBM modeling performance with increasing HR. The effect estimate was –0.0002 (95% CI –0.0003 to –0.0002) per percent point in HR, which in the most extreme case (ie, 100% HR) would only result in a decrease in AUROC of 0.02. Similarly, the TSTR from MLP was not affected by HR in most models (5/7, 71%). In adversarial random forest and robust tabular variational autoencoder, there was a significant negative association with, again, very small effect estimates (OR –0.0001, 95% CI –0.0002 to –0.0001 and OR –0.0001, 95% CI –0.0001 to 0.0000, respectively). Consistent with these findings, the variance explained by the fixed effect was negligible across all SDG models, and in most models, the random effect explained most of the variance (Table 4).

**Table 4:** Modeling the effect of hallucination rate (HR) on the downstream performance^a^.

SDG^b^ model and AI^c^ and ML^d^ model		Fixed effect HR, OR^e^ (95% CI)	*P* value	*R*^2^ (fixed effect)	*R*^2^ (overall)
**ST^f^**					
	LGBM^g^	0.0000 (–0.0001 to 0.0001)	.70	0.0000	0.9962
	MLP^h^	–0.0001 (–0.0001 to 0.0000)	.18	0.0003	0.9911
**BN^i^**					
	LGBM	0.0000 (–0.0001 to 0.0002)	.74	0.0000	0.9932
	MLP	0.0003 (0.0001 to 0.0005)	.10	0.0029	0.9662
**ARF^j^**					
	LGBM	–0.0001 (–0.0002 to 0.0000)	.15	0.0003	0.9985
	*MLP^k^*	–*0.0001* *(*–*0.0002* *to* –*0.0001)*	*.002*	*0.0007*	*0.9918*
**CTGAN^l^**					
	*LGBM*	–*0.0002* *(*–*0.0003* *to* *−0.0002)*	*<.001*	*0.0007*	*0.9905*
	MLP	–0.0001 (–0.0003 to 0.0002)	.70	0.0001	0.9866
**TVAE^m^**					
	LGBM	0.0004 (–0.0003 to 0.0011)	.40	0.0050	0.9778
	MLP	0.0000 (–0.0001 to 0.0001)	.80	0.0000	0.9784
**RTVAE^n^**					
	LGBM	0.0000 (−0.0002 to 0.0001)	.76	0.0000	0.9683
	*MLP*	–*0.0001 (*–*0.0001 to 0.0000)*	*.007*	*0.0003*	*0.9730*
**NFlow^o^**					
	LGBM	−0.0016 (−0.0038 to 0.0006)	.14	0.0675	0.0675
	MLP	0.0004 (−0.0015 to 0.0022)	.70	0.0049	0.0049

^a^Linear mixed-effect models were fitted for each synthetic data generation model separately, with the following number of observations: 1962 for light gradient boosted decision tree and 1964 for multilayer perceptron for sequential decision trees; 1354 (light gradient boosted decision tree and multilayer perceptron) for Bayesian network; 1354 (light gradient boosted decision tree and multilayer perceptron) for adversarial random forest; 1354 (light gradient boosted decision tree and multilayer perceptron) for conditional generative adversarial network; 1352 (light gradient boosted decision tree) and 1354 (multilayer perceptron) for tabular variational autoencoder; 1349 (light gradient boosted decision tree) and 1354 (multilayer perceptron) for robust tabular variational autoencoder; and 32 (light gradient boosted decision tree and multilayer perceptron) for normal flow. Health care populations were considered as random effects, HR as fixed effects, and the train synthetic test real as an outcome. Both light gradient boosted decision tree and multilayer perceptron are considered. The coefficients for the HR in percentages are indicated. We provide the variance explained (ie, *R*^2^) by the fixed effect only and by both fixed and marginal effects together (ie, *R*^2^ overall) for all models. For normal flow, there was no random effect since only one health care population met the requirements of HR range; therefore, *R*^2^ and *R*^2^ overall are identical.

^b^SDG: synthetic data generation.

^c^AI: artificial intelligence.

^d^ML: machine learning.

^e^OR: odds ratio.

^f^ST: sequential decision tree.

^g^LGBM: light gradient boosted decision tree.

^h^MLP: multilayer perceptron.

^i^BN: Bayesian network.

^j^ARF: adversarial random forest.

^k^Italicized text indicates models with *P*<.05.

^l^CTGAN: conditional generative adversarial network.

^m^TVAE: tabular variational autoencoder.

^n^RTVAE: robust tabular variational autoencoder.

^o^NFlow: normal flow.

**Figure 5:**
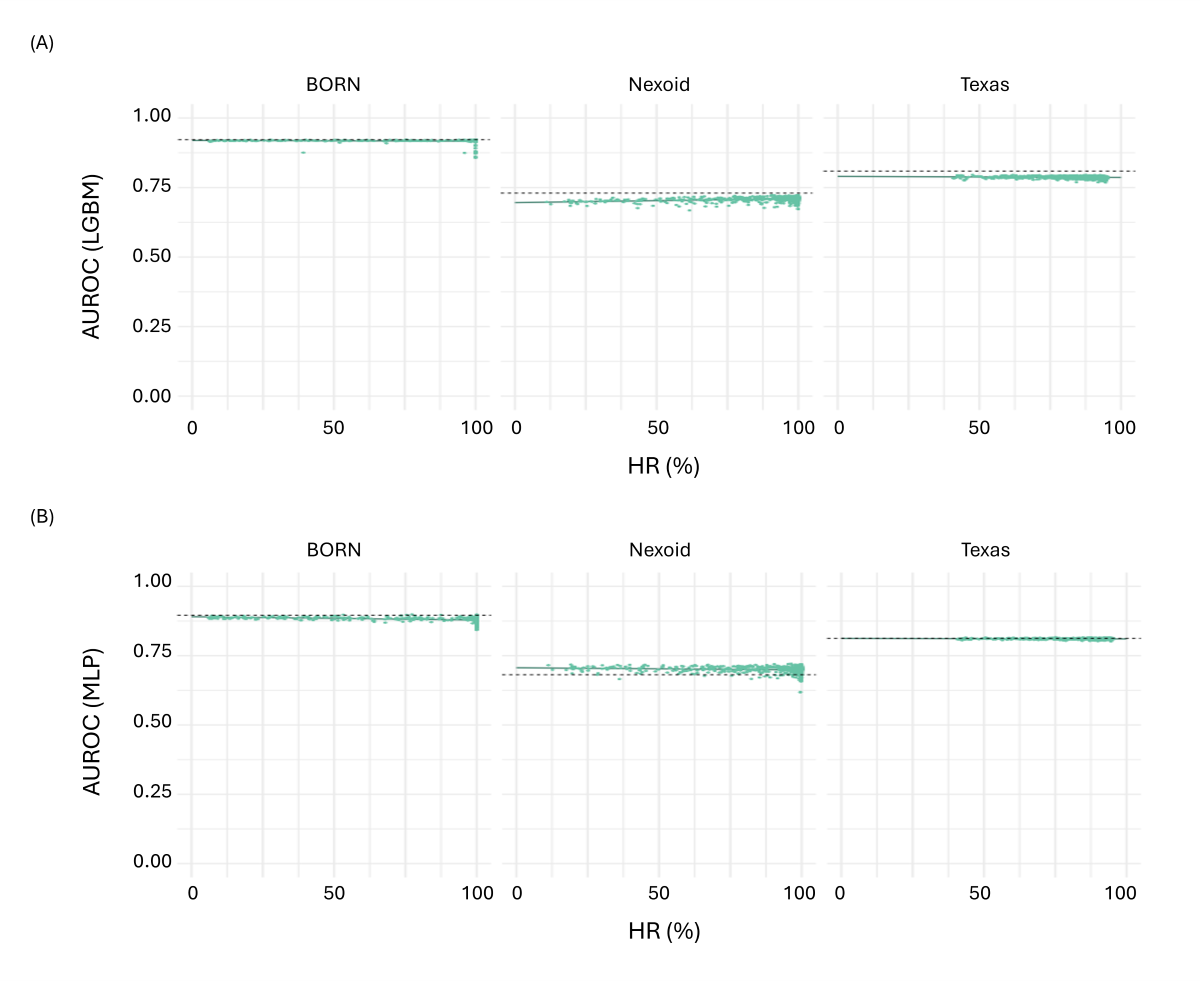
Mixed-effects model with health care population as a random effect, hallucination rate (HR) as a fixed effect, and train synthetic test real (TSTR) as an outcome for the synthetic data generation (SDG) model sequential decision trees (STs). HR in synthetic datasets was determined as described and averaged across the 10 synthetic datasets per trained SDG model. TSTR for a dataset was measured as the area under the receiver operating characteristic curve (AUROC) for light gradient boosted decision tree (LGBM) and multilayer perceptron (MLP) models. The green line is the predicted AUROC by the mixed-effects model, while the points are the observed AUROC. The dashed gray line is the AUROC by train real test real (TRTR) when being trained on real data.

In Figure 5, the prognostic AI and ML model performance trend for the SDG model ST (the example shown in Figure 4) is illustrated across the different populations. ST generated synthetic variants only for Better Outcomes Registry & Network, Nexoid, and Texas, with sufficient spread in the HR across variants. Results for the LGBM and the MLP models are presented. As shown in Table 4, this effect was similar for the other SDG models.

Again, these analyses were repeated with fewer population variants (ie, a 50% and 25% subset). While the prognostic AI and ML model performance across all downstream tasks was similar to the ones shown in Table 3, the estimated effect for the HR on downstream performance had slight differences. More importantly, the smaller-scale evaluations reduced the number of populations with sufficient spread in HR for modeling, with a resulting sparse coverage in the random effect.

## Discussion

### Principal Findings

In this study, we examined hallucinations in synthetic tabular health data. In total, 12 large datasets were used in a simulation of the relationship between dataset complexity and the HR and the HR and the downstream binary prediction performance of the generated datasets.

Our findings suggest that hallucinations can be very common in synthetic tabular health data and, as hypothesized in the Introduction section, depend on the dataset’s complexity. However, evidence from this study did not support the second hypothesis that the greater the rate of hallucinations, the less effective the prognostic models would be. This means that prognostic AI and ML modeling was not negatively (or positively) affected by increasing hallucinations in most cases. In those cases, where a negative trend was observed, this trend was negligibly small.

### Comparison to Prior Work

To our knowledge, hallucinations in tabular synthetic data have not been systematically studied yet. While previous work on evaluating tabular synthetic data focused on utility, privacy, and fairness [31,32] without explicitly investigating hallucinations, this phenomenon has received considerable attention in generative text modeling. In this modality, hallucinations are typically seen as a major limitation [13-15].

Intuitively, hallucinated tabular data can also pose limitations with the potential to degrade the performance of a prognostic AI and ML model because the model would learn patterns that are nonexistent in the population it is deployed on. However, our findings suggest that this is not the case.

One potential explanation is that hallucinations may be mainly driven by statistically independent variables that are not associated with the outcome and thus less relevant for prognostic AI and ML modeling. If synthetic records have an invalid combination of values for such variables, they are hallucinated but can still preserve valid combinations of values for variables that are relevant to prognostic modeling. In addition, high-cardinality variables may have long-tailed distributions, meaning that some categories are very rare. Hallucinations that affect these rare categories would contribute little to the overall predictive performance: If the prediction algorithm does not learn these rare (hallucinated) values because they are in the long tail, then the impact on predictions on unseen data will be minimal. If it does memorize them, the impact will still be minimal as these specific values are unlikely to appear in unseen data.

While hallucinations may not impact AI and ML modeling performance, their negative perception in previous work offers an important insight; they can still have a nontrivial impact on the trust in and acceptance of SDG by clinicians and researchers. In a sensitive sector such as health care, trust has been shown to be crucial for technology adoption [129]. In the context of trust, hallucinated records that violate real-world constraints, such as, female patients with prostate cancer or a young adult with a residency in a retirement home seem more severe than hallucinated patients that do not exist in a certain population but are, in theory, plausible patients (eg, a male patient with breast cancer). Fidelity metrics based on marginal or multivariate distributions are not designed to detect such violations. This means, as part of a trust-building exercise, it would be very valid and important to check synthetic datasets for such obvious real-world constraints, although they do not necessarily impact prognostic AI and ML model performance.

### Strengths and Limitations

This study explored hallucinations in synthetic health care data and their impact on prognostic AI and ML model performance. To our knowledge, this is the first study investigating hallucinations in tabular synthetic data in a large-scale methodological setup, including 6354 SDG training datasets derived from 12 real-world health care reference populations, 7 state-of-the-art SDG models, and 2 widely used prognostic AI and ML models as downstream tasks. Secondary analyses using only 50% and 25% of the population variants suggest that smaller-scale designs may be feasible when the population variants exhibit sufficient spread in the HR to detect meaningful trends.

Nevertheless, there are some limitations to highlight.

First, our definition of hallucinations provided one implementation of the concept of *factuality*. However, there may be other approaches for defining hallucinated records in synthetic data. For example, another option would be to search for violations of real-world constraints as mentioned previously (eg, prostate cancer in female patients), which could be described as hallucinations based on clinical plausibility. We decided not to rely on such a definition for two reasons: (1) the definition of real-world constraints requires a high degree of domain expertise specific to each dataset and (2) such implausible records would be a subset of nonexistent records. Our definition was consequently broader, capturing implausible records as well as other nonexistent records. Another definition could allow for or focus more on the distribution than on record-level similarity (ie, hallucination as distribution shift or based upon probabilistic similarity). Again, this is very likely a less sensitive definition in that it does not label nonexistent records as hallucinated, provided they match the underlying distribution. Hallucinations may also be defined in terms of statistical associations or patterns whereby a substantially different (ie, stronger or weaker) association can be considered a hallucination. In addition, such definitions typically require the specification of a threshold that would be hard to justify and further complicate interpretation.

Second, the choice of discretization in the implementation of our definition of hallucinations is ultimately dataset dependent and was informed by domain knowledge. In health care data, divergences in categorical versus numerical variables carry fundamentally different interpretations that should be accounted for in a distance-based definition of hallucinations. However, it must be noted that the number of bins can change the identification of hallucinations, with more bins increasing sensitivity and fewer bins introducing more tolerance with the risk of underdetection. In addition, records with values at the boundary of the discretization bin could be misclassified as hallucinations. While this effect was low in our scenario, where datasets were primarily categorical and the number of datasets under investigation was large (Multimedia Appendix 1), such an implementation could inflate the HR.

Third, any definition of hallucination based on violations of factuality, as the one in this study and those described previously, requires access to ground truth or population data. This dependency makes it difficult to evaluate the HR for a specific synthetic dataset, as the population data are often not readily available. However, if hallucinations are conceptualized as substantially different statistical associations, then the replicability of population parameters may offer an operationalizable way to quantify hallucinations and is, in fact, a utility metric that is used in certain synthetic data use cases [130].

Fourth, the population variants used in this study were predominantly of higher complexity, with relatively few examples of low-complexity data. Therefore, the findings may be more representative of scenarios involving high-cardinality or high-dimensional data. However, these datasets are commonly used in clinical research, supporting the relevance of our findings to many real-world health care research scenarios. In addition, while the sampling of population variants was necessary to manage the large combinatory space, the sampled variants may not be representative of the entire combinatory space.

Fifth, the downstream task under investigation was prognostic AI and ML modeling measured as AUROC. We applied 5-fold cross-validation to set hyperparameters for LGBM but did not perform exhaustive hyperparameter tuning for MLP. The default MLP settings already resulted in a performance that was comparable to and sometimes even outperformed LGBM, so that a different setup of hyperparameters was unlikely to relevantly improve performance, and we refrained from hyperparameter tuning for MLP. However, it may be valuable in other datasets.

Finally, we were interested in prognostic AI and ML modeling. However, SDG has also been proposed as a privacy-enhancing technology in the context of clinical trials [131]. Such a use case may be more sensitive to hallucinations if, for example, an external control arm is propensity score matched against the intervention arm. In contrast, descriptive statistics, particularly marginal distributions, are very likely not affected by hallucinations. Ultimately, however, it remains unclear at this stage which downstream tasks are most sensitive to hallucinated records, and their impact on specific use cases is speculative. Further systematic research is needed to identify which types of analyses are most vulnerable to hallucinations in synthetic tabular data.

## Appendix

Multimedia Appendix 1 Data descriptions, supplemental methods, and supplemental results.

## Abbreviations

AI: artificial intelligence
AUROC: area under the receiver operating characteristic curve
HR: hallucination rate
LGBM: light gradient boosted decision tree
LLM: large language model
ML: machine learning
MLP: multilayer perceptron
OR: odds ratio
REB: Research Ethics Board
SDG: synthetic data generation
ST: sequential decision tree
TSTR: train synthetic test real

## Data Availability

Some datasets analyzed during this study are publicly available; some datasets analyzed during this study are not publicly available due to privacy. Details about data availability are provided in Multimedia Appendix 1 for each population. All original code for our analysis has been deposited in the Open Science Framework [132].

## References

[1] Baker S, Kanade T (2000). Hallucinating faces. Proceedings 4th IEEE International Conference on Automatic Face and Gesture Recognition.

[2] Zhang K, Zhang Z, Cheng C (2018). Super-identity convolutional neural network for face hallucination. Proceedings of the 15th European Conference on Computer Vision.

[3] Wang H, Chi J, Li X, Wu C, Wu H (2023). Generative facial prior embedded degradation adaption network for heterogeneous face hallucination. Multimed Tools Appl.

[4] Huang H, He R, Sun Z, Tan T (2019). Wavelet domain generative adversarial network for multi-scale face hallucination. Int J Comput Vis.

[5] Zhang Y, Tsang IW, Li J, Liu P, Lu X, Yu X (2021). Face hallucination with finishing touches. IEEE Trans Image Process.

[6] Marnerides D, Bashford-Rogers T, Debattista K (2021). Deep HDR hallucination for inverse tone mapping. Sensors (Basel).

[7] Li M, Sun Y, Zhang Z, Xie H, Yu J (2019). Deep learning face hallucination via attributes transfer and enhancement. Proceedings of the 2019 IEEE International Conference on Multimedia and Expo.

[8] Zhang Y, Yu X, Lu X, Liu P (2022). Pro-UIGAN: progressive face hallucination from occluded thumbnails. IEEE Trans Image Process.

[9] Shao WZ, Xu JJ, Chen L, Ge Q, Wang L, Bao B, Li H (2019). On potentials of regularized Wasserstein generative adversarial networks for realistic hallucination of tiny faces. Neurocomputing.

[10] Shao WZ, Xu JJ, Chen L, Ge Q, Wang LQ, Bao BK, Li HB (2019). Tiny face hallucination via boundary equilibrium generative adversarial networks. Proceedings of the 10th International Conference on Graphics and Image Processing.

[11] Shao W, Zhang M, Li H (2021). Tiny face hallucination via relativistic adversarial learning. J Electron Inf Technol.

[12] Ji Z, Lee N, Frieske R, Yu T, Su D, Xu Y, Ishii E, Bang YJ, Madotto A, Fung P (2023). Survey of hallucination in natural language generation. ACM Comput Surv.

[13] Asgari E, Montaña-Brown N, Dubois M, Khalil S, Balloch J, Yeung JA, Pimenta D (2025). A framework to assess clinical safety and hallucination rates of LLMs for medical text summarisation. NPJ Digit Med.

[14] Maddox T, Babski D, Embi P, Gerhart J, Goldsack J, Parikh R, Sarich T (2025). Generative Artificial Intelligence in Health and Medicine: Opportunities and Responsibilities for Transformative Innovation.

[15] Vrdoljak J, Boban Z, Vilović M, Kumrić M, Božić J (2025). A review of large language models in medical education, clinical decision support, and healthcare administration. Healthcare (Basel).

[16] Lee P, Goldberg C, Kohane I (2023). The AI revolution in medicine: GPT-4 and beyond.

[17] Bent AA (2023). Large language models: AI's legal revolution. Pace L Rev.

[18] Tan J, Westermann H, Benyekhlef K (2023). ChatGPT as an artificial lawyer?. Proceedings of the 2023 International Conference and Workshop on Artificial Intelligence.

[19] Alkaissi H, McFarlane S (2023). Artificial hallucinations in ChatGPT: implications in scientific writing. Cureus.

[20] Athaluri SA, Manthena SV, Kesapragada VS, Yarlagadda V, Dave T, Duddumpudi RT (2023). Exploring the boundaries of reality: investigating the phenomenon of artificial intelligence hallucination in scientific writing through ChatGPT references. Cureus.

[21] Sharun K, Banu SA, Pawde AM, Kumar R, Akash S, Dhama K, Pal A (2023). ChatGPT and artificial hallucinations in stem cell research: assessing the accuracy of generated references - a preliminary study. Ann Med Surg (Lond).

[22] Proctor J B.C. lawyer reprimanded for citing fake cases invented by ChatGPT. CBC News.

[23] Geroimenko V, Geroimenko V (2025). Generative AI hallucinations in healthcare: a challenge for prompt engineering and creativity. Human-Computer Creativity: Generative AI in Education, Art, and Healthcare.

[24] Vishwanath PR, Tiwari S, Naik TG, Gupta S, Thai DN Faithfulness hallucination detection in healthcare AI. OpenReview.

[25] Kim Y, Jeong H, Chen S, Li SS, Lu M, Alhamoud K, Mun J, Grau C Medical hallucinations in foundation models and their impact on healthcare. arXiv.

[26] Walonoski J, Kramer M, Nichols J, Quina A, Moesel C, Hall D, Duffett C, Dube K, Gallagher T, McLachlan S (2018). Synthea: an approach, method, and software mechanism for generating synthetic patients and the synthetic electronic health care record. J Am Med Inform Assoc.

[27] Jeanson F, Farkouh ME, Godoy LC, Minha S, Tzuman O, Marcus G (2024). Medical calculators derived synthetic cohorts: a novel method for generating synthetic patient data. Sci Rep.

[28] Al-Dhamari I, Abu Attieh H, Prasser F (2024). Synthetic datasets for open software development in rare disease research. Orphanet J Rare Dis.

[29] Templ M, Meindl B, Kowarik A, Dupriez O (2017). Simulation of synthetic complex data: the R package simPop. J Stat Soft.

[30] Rineer J, Kruskamp N, Kery C, Jones K, Hilscher R, Bobashev G (2025). A national synthetic populations dataset for the United States. Sci Data.

[31] Kaabachi B, Despraz J, Meurers T, Otte K, Halilovic M, Kulynych B, Prasser F, Raisaro JL (2025). A scoping review of privacy and utility metrics in medical synthetic data. NPJ Digit Med.

[32] Vallevik VB, Babic A, Marshall SE, Elvatun S, Brøgger HM, Alagaratnam S, Edwin B, Veeraragavan NR, Befring AK, Nygård JF (2024). Can I trust my fake data - a comprehensive quality assessment framework for synthetic tabular data in healthcare. Int J Med Inform.

[33] El Emam K (2020). Seven ways to evaluate the utility of synthetic data. IEEE Secur Privacy.

[34] El Emam K, Mosquera L, Fang X, El-Hussuna A (2022). Utility metrics for evaluating synthetic health data generation methods: validation study. JMIR Med Inform.

[35] Kaabachi B, Despraz J, Meurers T Can we trust synthetic data in medicine? A scoping review of privacy and utility metrics. medRxiv.

[36] Maynez J, Narayan S, Bohnet B (2020). On faithfulness and factuality in abstractive summarization. Proceedings of the 58th Annual Meeting of the Association for Computational Linguistics.

[37] Lee M (2023). A mathematical investigation of hallucination and creativity in GPT models. Mathematics.

[38] Chen PH, Liu Y, Peng L (2019). How to develop machine learning models for healthcare. Nat Mater.

[39] An Q, Rahman S, Zhou J, Kang JJ (2023). A comprehensive review on machine learning in healthcare industry: classification, restrictions, opportunities and challenges. Sensors (Basel).

[40] Kadra A, Lindauer M, Hutter F, Grabocka J Well-tuned simple nets excel on tabular datasets. arXiv.

[41] Valero De Bernabé J, Soriano T, Albaladejo R, Juarranz M, Calle ME, Martínez D, Domínguez-Rojas V (2004). Risk factors for low birth weight: a review. Eur J Obstet Gynecol Reprod Biol.

[42] Yadav DK, Chaudhary U, Shrestha N (2011). Risk factors associated with low birth weight. J Nepal Health Res Counc.

[43] HCUP State Inpatient Databases (SID). Healthcare Cost and Utilization Project (HCUP). Agency for Healthcare Research and Quality.

[44] França UL, McManus ML (2018). Frequency, trends, and antecedents of severe maternal depression after three million U.S. births. PLoS One.

[45] Brownlee SA, Blackwell RH, Blanco BA, Zapf MA, Kliethermes S, Gupta GN, Kuo PC, Kothari AN (2017). Impact of post-hospital syndrome on outcomes following elective, ambulatory surgery. Ann Surg.

[46] Maclagan LC, Park J, Sanmartin C, Mathur KR, Roth D, Manuel DG, Gershon A, Booth GL, Bhatia S, Atzema CL, Tu JV (2014). The CANHEART health index: a tool for monitoring the cardiovascular health of the Canadian population. CMAJ.

[47] Berry I, O'Neill M, Sturrock SL, Wright JE, Acharya K, Brankston G, Harish V, Kornas K, Maani N, Naganathan T, Obress L, Rossi T, Simmons AE, Van Camp M, Xie X, Tuite AR, Greer AL, Fisman DN, Soucy JP (2021). A sub-national real-time epidemiological and vaccination database for the COVID-19 pandemic in Canada. Sci Data.

[48] Marwitz K, Jones SC, Kortepeter CM, Dal Pan GJ, Muñoz MA (2020). An evaluation of postmarketing reports with an outcome of death in the US FDA adverse event reporting system. Drug Saf.

[49] Meddings J, Reichert H, Smith SN, Iwashyna TJ, Langa KM, Hofer TP, McMahon LF (2017). The impact of disability and social determinants of health on condition-specific readmissions beyond medicare risk adjustments: a cohort study. J Gen Intern Med.

[50] Johnson A, Pollard T, Mark R MIMIC-III clinical database CareVue subset (version 1.4). PhysioNet.

[51] Johnson AE, Pollard TJ, Shen L, Lehman LW, Feng M, Ghassemi M, Moody B, Szolovits P, Celi LA, Mark RG (2016). MIMIC-III, a freely accessible critical care database. Sci Data.

[52] Goldberger AL, Amaral L, Glass L, Hausdorff JM, Ivanov PC, Mark RG, Mietus JE, Moody GB, Peng C, Stanley HE (2000). PhysioBank, PhysioToolkit, and PhysioNet. Circulation.

[53] Pishgar M, Theis J, Del Rios M, Ardati A, Anahideh H, Darabi H (2022). Prediction of unplanned 30-day readmission for ICU patients with heart failure. BMC Med Inform Decis Mak.

[54] Aliu O, Auger KA, Sun GH, Burke JF, Cooke CR, Chung KC, Hayward RA (2014). The effect of pre-Affordable Care Act (ACA) medicaid eligibility expansion in New York State on access to specialty surgical care. Med Care.

[55] Kahn JM, Le T, Angus DC, Cox CE, Hough CL, White DB, Yende S, Carson SS, ProVent Study Group Investigators (2015). The epidemiology of chronic critical illness in the United States*. Crit Care Med.

[56] Sabbatini AK, Kocher KE, Basu A, Hsia RY (2016). In-hospital outcomes and costs among patients hospitalized during a return visit to the emergency department. JAMA.

[57] Grantham J COVID-19 survival calculator. Nexoid.

[58] Texas hospital inpatient discharge public use data file. Texas Department of State Health Services.

[59] Zhang J, Yu P (2023). Machine learning methods for prediction of COVID-19 patient length of stay: using Texas PUDF data. Proceedings of the 3rd International Conference on Electrical, Computer, Communications and Mechatronics Engineering.

[60] Goss LB, Ortiz JR, Okamura DM, Hayward K, Goss CH (2015). Significant reductions in mortality in hospitalized patients with systemic lupus erythematosus in Washington State from 2003 to 2011. PLoS One.

[61] Metcalfe D, Zogg CK, Haut ER, Pawlik TM, Haider AH, Perry DC (2019). Data resource profile: state inpatient databases. Int J Epidemiol.

[62] Barrett ML, Wier LM, Jiang HJ, Steiner CA All-cause readmissions by payer and age, 2009–2013. Healthcare Cost and Utilization Project (HCUP) Statistical Briefs.

[63] Emam KE, Kababji SE, Pilgram L, Cano V, Liu D pysdg. Open Science Framework.

[64] Hothorn T, Hornik K, Zeileis A (2006). Unbiased recursive partitioning: a conditional inference framework. J Comput Graph Stat.

[65] Read J, Pfahringer B, Holmes G, Frank E (2009). Classifier chains for multi-label classification. Proceedings of the 2009 Conference on Machine Learning and Knowledge Discovery in Databases.

[66] Drechsler J, Reiter Jp (2011). An empirical evaluation of easily implemented, nonparametric methods for generating synthetic datasets. Comput Stat Data Anal.

[67] Arslan RC, Schilling KM, Gerlach TM, Penke L (2021). Using 26,000 diary entries to show ovulatory changes in sexual desire and behavior. J Pers Soc Psychol.

[68] Bonnéry D, Feng Y, Henneberger AK, Johnson TL, Lachowicz M, Rose BA, Shaw T, Stapleton LM, Woolley ME, Zheng Y (2019). The promise and limitations of synthetic data as a strategy to expand access to state-level multi-agency longitudinal data. J Res Educ Eff.

[69] Sabay A, Harris L, Bejugama V, Jaceldo-Siegl K (2018). Overcoming small data limitations in heart disease prediction by using surrogate data. SMU Data Sci Rev.

[70] Formal privacy and synthetic data for the American community survey. US Census Bureau.

[71] Utility of synthetic microdata generated using tree-based methods. United Nations Economic Commission for Europe.

[72] Raab GM, Nowok B, Dibben C (2016). Practical data synthesis for large samples. J Priv Confid.

[73] Nowok B, Raab GM, Dibben C (2017). Providing bespoke synthetic data for the UK Longitudinal Studies and other sensitive data with the synthpop package for R1. Stat J IAOS.

[74] Quintana DS (2020). A synthetic dataset primer for the biobehavioural sciences to promote reproducibility and hypothesis generation. Elife.

[75] Kaur D, Sobiesk M, Patil S, Liu J, Bhagat P, Gupta A, Markuzon N (2021). Application of Bayesian networks to generate synthetic health data. J Am Med Inform Assoc.

[76] Murphy KP (2012). Machine Learning: A Probabilistic Perspective.

[77] Qian Z, Cebere BC, van der Schaar M Synthcity: facilitating innovative use cases of synthetic data in different data modalities. arXiv.

[78] Goodfellow IJ, Pouget-Abadie J, Mirza M, Xu B, Warde-Farley D, Ozair S, Courville A, Bengio Y Generative adversarial networks. arXiv.

[79] Bourou S, El Saer A, Velivassaki T, Voulkidis A, Zahariadis T (2021). A review of tabular data synthesis using GANs on an IDS dataset. Information.

[80] Kingma DP, Welling M Auto-encoding variational bayes. arXiv.

[81] Wan Z, Zhang Y, He H (2017). Variational autoencoder based synthetic data generation for imbalanced learning. Proceedings of the 2017 IEEE Symposium Series on Computational Intelligence.

[82] Ishfaq H, Hoogi A, Rubin D TVAE: triplet-based variational autoencoder using metric learning. arXiv.

[83] Sohn K, Lee H, Yan X (2015). Learning structured output representation using deep conditional generative models. Proceedings of the 29th International Conference on Neural Information Processing Systems.

[84] Salim Jr A Synthetic patient generation: a deep learning approach using variational autoencoders. arXiv.

[85] Akrami H, Joshi AA, Li J, Aydöre S, Leahy RM (2022). A robust variational autoencoder using beta divergence. Knowl Based Syst.

[86] Snoek J, Larochelle H, Adams RP (2012). Practical Bayesian optimization of machine learning algorithms. Proceedings of the 25th International Conference on Neural Information Processing Systems.

[87] Bartz E, Bartz-Beielstein T, Zaefferer M, Mersmann O (2023). Hyperparameter Tuning for Machine and Deep Learning with R: A Practical Guide.

[88] Bischl B, Binder M, Lang M, Pielok T, Richter J, Coors S, Thomas J Hyperparameter optimization: foundations, algorithms, best practices and open challenges. arXiv.

[89] Binder M, Pfisterer F, Bischl B (2020). Collecting empirical data about hyperparameters for data driven AutoML. Proceedings of the 7th ICML Workshop on Automated Machine Learning.

[90] Kühn D, Probst P, Thomas J, Bischl B Automatic exploration of machine learning experiments on OpenML. arXiv.

[91] Juwara L, El-Hussuna A, El Emam K (2024). An evaluation of synthetic data augmentation for mitigating covariate bias in health data. Patterns (N Y).

[92] Huang Y, Li W, Macheret F, Gabriel RA, Ohno-Machado L (2020). A tutorial on calibration measurements and calibration models for clinical prediction models. J Am Med Inform Assoc.

[93] Kull M, Filho TS, Flach P (2017). Beta calibration: a well-founded and easily implemented improvement on logistic calibration for binary classifiers. Proceedings of the 20th International Conference on Artificial Intelligence and Statistics.

[94] El Emam K sdgm package. Open Science Framework.

[95] TensorFlow for R - reference. R Studio.

[96] Ruíz JS, López OA, Ramírez GH, Hiriart JC, Ruíz JS, López OA, Ramírez GH, Hiriart JC (2023). Generalized linear mixed models for proportions and percentages. Generalized Linear Mixed Models with Applications in Agriculture and Biology.

[97] Bates D, Mächler M, Bolker B, Walker S (2015). Fitting linear mixed-effects models using lme4. J Stat Softw.

[98] Kuznetsova A, Brockhoff PB, Christensen RH (2017). lmerTest package: tests in linear mixed effects models. J Stat Softw.

[99] MuMIn: multi-model inference. Cran R.

[100] Cano JR (2013). Analysis of data complexity measures for classification. Expert Syst Appl.

[101] Lorena AC, Garcia LP, Lehmann J, Souto MC, Ho TK (2019). How complex is your classification problem?. ACM Comput Surv.

[102] El Emam K, Mosquera L, Zheng C (2021). Optimizing the synthesis of clinical trial data using sequential trees. J Am Med Inform Assoc.

[103] Ankan A, Panda A (2015). pgmpy: probabilistic graphical models using Python. Proceedings of the 14th Python in Science Conference.

[104] Xu L, Skoularidou M, Cuesta-Infante A, Veeramachaneni K Modeling tabular data using conditional GAN. arXiv.

[105] Watson DS, Blesch K, Kapar J, Wright MN Adversarial random forests for density estimation and generative modeling. arXiv.

[106] Durkan C, Bekasov A, Murray I, Papamakarios G Neural spline flows. arXiv.

[107] Liu D, Kababji SE, Mitsakakis N, Pilgram L, Walters T, Clemons M, Pond G, El-Hussuna A, Emam KL Synthetic data generation for augmenting small samples. arXiv.

[108] Wickham H, François R, Henry L, Müller K, Vaughan D dplyr: a grammar of data manipulation. dplyr.

[109] Hyland SL, Esteban C, Rätsch G Real-valued (medical) time series generation with recurrent conditional GANs. arXiv.

[110] Kushwaha PK, Kumaresan M (2021). Machine learning algorithm in healthcare system: a review. Proceedings of the 2021 International Conference on Technological Advancements and Innovations.

[111] Gupta S, Sedamkar RR, Jain V, Chatterjee JM (2020). Machine learning for healthcare: introduction. Machine Learning with Health Care Perspective: Machine Learning and Healthcare.

[112] Andaur Navarro CL, Damen JA, van Smeden M, Takada T, Nijman SW, Dhiman P, Ma J, Collins GS, Bajpai R, Riley RD, Moons KG, Hooft L (2023). Systematic review identifies the design and methodological conduct of studies on machine learning-based prediction models. J Clin Epidemiol.

[113] Rousset A, Dellamonica D, Menuet R, Lira Pineda A, Sabatine MS, Giugliano RP, Trichelair P, Zaslavskiy M, Ricci L (2022). Can machine learning bring cardiovascular risk assessment to the next level? A methodological study using FOURIER trial data. Eur Heart J Digit Health.

[114] Weng SF, Reps J, Kai J, Garibaldi JM, Qureshi N (2017). Can machine-learning improve cardiovascular risk prediction using routine clinical data?. PLoS One.

[115] Akyea RK, Qureshi N, Kai J, Weng SF (2020). Performance and clinical utility of supervised machine-learning approaches in detecting familial hypercholesterolaemia in primary care. NPJ Digit Med.

[116] Desai RJ, Wang SV, Vaduganathan M, Evers T, Schneeweiss S (2020). Comparison of machine learning methods with traditional models for use of administrative claims with electronic medical records to predict heart failure outcomes. JAMA Netw Open.

[117] Li Y, Jiang L, He J, Jia K, Peng Y, Chen M (2020). Machine learning to predict the 1-year mortality rate after acute anterior myocardial infarction in Chinese patients. Ther Clin Risk Manag.

[118] Shwartz-Ziv R, Armon A (2022). Tabular data: deep learning is not all you need. Inf Fusion.

[119] Grinsztajn L, Oyallon E, Varoquaux G Why do tree-based models still outperform deep learning on typical tabular data?. arxiv.

[120] Van Calster B, Collins GS, Vickers AJ, Wynants L, Kerr KF, Barreñada L, Varoquaux G, Singh K, Moons KG Performance evaluation of predictive AI models to support medical decisions: Overview and guidance. arXiv.

[121] Bradshaw TJ, Huemann Z, Hu J, Rahmim A (2023). A guide to cross-validation for artificial intelligence in medical imaging. Radiol Artif Intell.

[122] Nakagawa S, Schielzeth H (2012). A general and simple method for obtaining R2 from generalized linear mixed-effects models. Methods Ecol Evol.

[123] (2018). Tri-council policy statement: ethical conduct for research involving humans – TCPS 2 (2022). Government of Canada.

[124] Dixon JR (1998). The international conference on harmonization good clinical practice guideline. Qual Assur.

[125] Guidance document: part C, division 5 of the food and drug regulations “drugs for clinical trials involving human subjects” (GUI-0100) - summary. Government of Canada.

[126] (2025). Natural health products regulations SOR/2003-196. Government of Canada.

[127] (2025). Medical devices regulations SOR/98-282. Government of Canada.

[128] Personal health information protection act, 2004, S.O. 2004, c. 3, Sched. A. Government of Ontario.

[129] van Hoorn R On the acceptance, adoption, and utility of synthetic data for healthcare innovation. Eindhoven University of Technology.

[130] El Emam K, Mosquera L, Fang X, El-Hussuna A (2024). An evaluation of the replicability of analyses using synthetic health data. Sci Rep.

[131] El Kababji S, Mitsakakis N, Fang X, Beltran-Bless A, Pond G, Vandermeer L, Radhakrishnan D, Mosquera L, Paterson A, Shepherd L, Chen B, Barlow WE, Gralow J, Savard M, Clemons M, El Emam K (2023). Evaluating the utility and privacy of synthetic breast cancer clinical trial data sets. JCO Clin Cancer Inform.

[132] Pilgram L, El Emam K Hallucinations in tabular synthetic data. Open Science Framework.

