# Supplementary material for: Magnitude and Impact of Hallucinations in Tabular Synthetic Health Data on Prognostic Machine Learning Models: Validation Study
Source: J Med Internet Res. 2025 Aug 18;27:e77893. doi: 10.2196/77893 (PMC12402739; doi:10.2196/77893)
Supplement: Multimedia Appendix 1 [file jmir_v27i1e77893_app1.pdf]

Magnitude and Impact of Hallucinations in Tabular Synthetic Health Data on Prognostic Machine Learning Models: Validation Study

Appendix A

Lisa Pilgram[1,2,3], Samer El Kababji[2], Dan Liu[1,2], Khaled El Emam[1,2]

[1]School of Epidemiology and Public Health, University of Ottawa, Ontario, Canada
[2]Children's Hospital of Eastern Ontario Research Institute, Ontario, Canada
[3]Department of Nephrology and Medical Intensive Care, Charité - Universitaetsmedizin Berlin, Berlin, Germany,

Corresponding Author:
Khaled El Emam
CHEO Research Institute
401 Smyth Road
Ottawa, Ontario K1H 8L1
Canada
kelemam@ehealthinformation.ca

Table of Contents

Data and Downstream Tasks 3
Better Outcomes Registry & Network (BORN) 3
California State Hospital Discharge Data 4
Canadian Community Health Survey (CCHS) 4
Canadian COVID-19 Data 5
FDA Adverse Event Reporting System (FAERS) 5
Florida State Hospital Discharge Data 6
Medical Information Mart for Intensive Care III (MIMIC-III) 7
New York State Hospital Discharge Data 8
COVID-19 Survival Calculator Data (Nexoid) 9
Texas State Hospital Discharge Data 10
Washington State Hospital Discharge Data 2007 11
Washington State Hospital Discharge Data 2008 12
Supplemental Methods 13
Sampling Scheme to Create Population Variants 13
Discretization of Numerical Variables to Identify Hallucinations 15
Definition of Complexity 15
Synthetic Data Generation 16
Sequential Decision Trees 16
Bayesian Networks 16
Conditional Generative Adversarial Network 16
Variational Autoencoder 17
Adversarial Random Forests (ARF) 17
Normalizing Flows (NFlow) 17
Implementation of LGBM and MLP 17
Mixed-Effect Models 18
Supplemental Results 18
Impact of Discretization on the HR 18
Sensitivity Analysis: Effect of Complexity on HR with 50% and 25% of the Population Variants 24
Sensitivity Analysis: Effect of HR on TSTR with 50% and 25% of the Population Variants 26
Robustness of TRTR Over 10 Different Splits 31
References 33

Data and Downstream Tasks

Each population is described in the following including information about access and its specific downstream task. The downstream task ultimately determined the *core* variables of the respective healthcare population. This means that the variables provided in the following tables (the outcome variables plus its predictors) are identical to the *core* variables as mentioned in the main manuscript. The downstream task itself was defined a priori based on domain expertise, related literature and consultations with the data providers.

Better Outcomes Registry & Network (BORN)

The BORN collects Ontario's prescribed perinatal, newborn and child registry with the role of facilitating quality care for families across the province. It can be accessed through a data request at https://bornontario.ca/en/data/data.aspx.

Using data from the BORN, birth weight is predicted considering maternal health conditions, drug intake and gestational age [1], [2]. More precisely, the following variables are used in our analysis:

**Variable**	**Description**	**Data Type**
Birth weight	Birthweight below 2,500 grams	Binary
Gestational age	Time from the first day of the mothers last menstrual period until the date of delivery	Categorical
Maternal age	Maternal age at birth	Categorical
Maternal BMI	Maternal pre-pregnancy BMI	Categorical
Parity	Number of previous pregnancies	Categorical
Preterm birth	Number of previous preterm births	Categorical
Abortions	Number of previous abortions	Categorical
Smoking	Maternal smoking status	Binary
Alcohol	Alcohol exposure during pregnancy	Binary
Prenatal screening	Conduction of prenatal screening	Binary
Addiction	Maternal substance use disorder	Binary
Anxiety	Maternal anxiety disorder	Binary
Depression	Maternal depressive disorder	Binary
Diabetes	Maternal diabetes mellitus	Binary
Genetics	Maternal genetic condition	Binary
Cocaine drug	Cocaine exposure during pregnancy	Binary
Hallucinogens drug	Hallucinogen exposure during pregnancy	Binary
Opioids drug	Opioid exposure during pregnancy	Binary

Table S1. Variables for the downstream analysis. The outcome variable is highlighted in italics. Data type gives information how the variable was measured. BMI: body-mass index.

California State Hospital Discharge Data

The California dataset contains the patient's hospital 2008 discharge data from California, State Inpatient Databases (SID), Healthcare Cost and Utilization Project (HCUP), Agency for Healthcare Research and Quality [3], and is available for purchase at https://hcup-us.ahrq.gov/tech_assist/centdist.jsp.

Using data from the California Inpatient Database, length of stay is predicted considering patient's demographics, their principal diagnosis, comorbidities and undertaken procedures [4], [5]. More precisely, the following variables are used in our analysis:

Variable	Description	Data Type
Length of stay	Length of stay ≥ 3 days	Binary
Age	Patient's age in years	Numerical
Gender	Patient's gender	Binary
Race	Patient's race	Categorical
Weekend admission	Admission on a weekend	Binary
DRG	Diagnostic Related Group for prospective payment purposes	Categorical
ICD-9	ICD-9 Diagnosis Code for principal diagnosis	Categorical
Chronic condition	Chronic condition of principal diagnosis	Binary
Body system	Body system affected by a chronic condition	Categorical
Procedure class	ICD Procedure Classes (e.g. minor diagnostic)	Categorical
Alcohol abuse	Alcohol abuse as comorbidity	Binary
Depression	Depressive disorder as comorbidity	Binary
Hypertension	Hypertension as comorbidity	Binary
Obesity	Obesity as comorbidity	Binary
Primary payer	Expected primary payer (e.g., Medicare)	Categorical

Table S2. Variables for the downstream analysis. The outcome variable is highlighted in italics. Data type gives information how the variable was measured. DRG: Diagnostic Related Group; ICD: International Classification of Disease.

Canadian Community Health Survey (CCHS)

The CCHS data are Canadian population-level information concerning health status, health system utilization and health determinants collected by Statistics Canada through telephone survey. The availability of CCHS data is restricted and requires an access request at https://www150.statcan.gc.ca/n1/pub/82-620-m/2005001/4144189-eng.htm.

Using data from the CCHS, cardiovascular risk indicated by the CANHEART Health Index is predicted considering patient's demographics and cardiovascular health factors [6]. More precisely, the following variables are used in our analysis:

Variable	Description	Data Type
Ideal CANHEART Index	CANHEART Index ≥ 3 indicating an ideal cardiovascular health status	Binary
Age	Patient's age in years	Categorical
Gender	Patient's gender	Binary
Education	Patient's highest level of education	Categorical
Marital status	Patient's marital status	Categorical
House income	Total household income from all sources	Categorical
Household size	Size of entire household	Categorical
Immigration	Whether a patient is an immigrant	Binary

Table S3. Variables for the downstream analysis. The outcome variable is highlighted in italics. Data type gives information how the variable was measured. The CANHEART Index is calculated from the following heath factors: smoking, obesity, hypertension, diabetes mellitus, physical activity and fruit and vegetable consumption. CANHEART Index: Cardiovascular Health in Ambulatory Care Research Team health index.

Canadian COVID-19 Data

The COVID-19 dataset collects Canadian health records of COVID-19 gathered by the Public Health Agency of Canada and is available at Esri Canada (https://resources-covid19canada.hub.arcgis.com/).

Using data from the Canadian COVID-19 dataset, case status is predicted considering patient's demographics, geographical factors and the time when the case occurred [7]. More precisely, the following variables are used in our analysis:

Variable	Description	Data Type
Deceased	Patient's status	Binary
Age	Patient's age in years	Categorical
Gender	Patient's gender	Binary
Date	Date when the case was reported	Datetime
Province	Province in Canada	Categorical
Exposure	Type of exposure	Categorical

Table S4. Variables for the downstream analysis. The outcome variable is highlighted in italics. Data type gives information how the variable was measured.

FDA Adverse Event Reporting System (FAERS)

The FAERS is a database comprising the information on adverse events and medication error reports submitted to FDA and can be downloaded at https://open.fda.gov/data/faers/.

Using data from the FAERS, death as outcome from an adverse event is predicted considering patient's demographics, the drug used and its indication [8]. More precisely, the following variables are used in our analysis:

**Variable**	**Description**	**Data Type**
Death	Patient's outcome from adverse event	Binary
Age	Patient's age in years	Numeric
Gender	Patient's gender	Categorical
Date	Date of the adverse event	Datetime
Weight	Patient's weight	Numeric
Drug	Drug used	Categorical
Indication	Indication for drug	Categorical

Table S5. Variables for the downstream analysis. The outcome variable is highlighted in italics. Data type gives information how the variable was measured.

Florida State Hospital Discharge Data

The Florida dataset contains the patient's hospital 2007 discharge data from Florida, State Inpatient Databases (SID), Healthcare Cost and Utilization Project (HCUP), Agency for Healthcare Research and Quality [3], and is available for purchase at https://hcup-us.ahrq.gov/tech_assist/centdist.jsp.

Using data from the Florida Inpatient Database, length of stay is predicted considering patient's demographics, their principal diagnosis and charges [4], [9]. More precisely, the following variables are used in our analysis:

Variable	Description	Data Type
Length of stay	Length of stay ≥ 3 days	Binary
Age	Patient's age in years	Numeric
Gender	Patient's gender	Binary
Race	Patient's race	Categorical
ZIP	Patient's zip code or living status (e.g. homeless)	Categorical
Admission type	Type of admission (e.g. emergency)	Categorical
Weekend admission	Admission on a weekend	Binary
DRG	Diagnostic Related Group for prospective payment purposes	Categorical
ICD-9	ICD-9 Diagnosis Code for principal diagnosis	Categorical
Primary payer	Expected primary payer (e.g., Medicare)	Categorical

Table S6. Variables for the downstream analysis. The outcome variable is highlighted in italics. Data type gives information how the variable was measured. DRG: Diagnostic Related Group; ICD: International Classification of Disease.

Medical Information Mart for Intensive Care III (MIMIC-III)

MIMIC-III is a large database that contains deidentified health-related data associated with over forty thousand patients who stayed in critical care units of the Beth Israel Deaconess Medical Center between 2001 and 2012 [10], [11]. The access to the MIMIC database is upon signing a data use agreement with PhysioNet at https://physionet.org/content/mimiciii/1.4/ [12].

Using data from the MIMIC-III database, readmission is predicted considering patient's demographics, vitals and laboratory parameters [13]. More precisely, the following variables are used in our analysis:

Variable	Description	Data Type
Readmission	*Readmission to Intensive Care Unit within 30 days after discharge*	*Binary*
Age	Patient's age in years	Numeric
Ethnicity	Patient's ethnicity group	Categorical
Admission type	Admission type (e.g. emergency)	Categorical
Heart rate	Heart rate at admission	Numeric
Systolic blood pressure	Systolic blood pressure at admission	Numeric
Diastolic blood pressure	Diastolic blood pressure at admission	Numeric
Respiratory rate	Respiratory rate at admission	Numeric
NT-proBNP	First laboratory test for N-terminal prohormone of brain natriuretic peptide after admission to ICU	Numeric
Creatinine	First laboratory test for serum creatinine after admission to ICU	Numeric
Blood urea nitrogen	First laboratory test for blood urea nitrogen after admission to ICU	Numeric
Potassium	First laboratory test for potassium after admission to ICU	Numeric
Cholesterol	First laboratory test for cholesterol after admission to ICU	Numeric

Table S7. Variables for the downstream analysis. The outcome variable is highlighted in italics. Data type gives information how the variable was measured.

New York State Hospital Discharge Data

The New York dataset contains the patient's hospital 2007 discharge data from New York, State Inpatient Databases (SID), Healthcare Cost and Utilization Project (HCUP), Agency for Healthcare Research and Quality [3], and is available for purchase at https://hcup-us.ahrq.gov/tech_assist/centdist.jsp.

Using data from the New York Inpatient Database, length of stay is predicted considering patient's demographics, their principal diagnosis and charge information [14], [15], [16]. More precisely, the following variables are used in our analysis:

Variable	Description	Data Type
Length of stay	Length of stay ≥ 3 days	Binary
Age	Patient's age in years	Numerical
Gender	Patient's gender	Binary
Race	Patient's race	Categorical
ZIP	Patient's zip code or living status (e.g. homeless)	Categorical
Admission type	Type of admission (e.g. emergency)	Categorical
Weekend admission	Admission on a weekend	Binary
DRG	Diagnostic Related Group for prospective payment purposes	Categorical
ICD-9	ICD-9 Diagnosis Code for principal diagnosis	Categorical
Chronic condition	Chronic condition of principal diagnosis	Binary
Body system	Body system affected by a chronic condition	Categorical
Procedure class	ICD Procedure Classes (e.g. minor diagnostic)	Categorical
Primary payer	Expected primary payer (e.g., Medicare)	Categorical

Table S8. Variables for the downstream analysis. The outcome variable is highlighted in italics. Data type gives information how the variable was measured. DRG: Diagnostic Related Group; ICD: International Classification of Disease.

COVID-19 Survival Calculator Data (Nexoid)

The COVID-19 survival dataset is a web-based survey data collected by a company called Nexoid in United Kingdom (UK). It is publicly available at https://www.covid19survivalcalculator.com/en/download.

Using data from the COVID-19 web-based survey (Nexoid), risk of infection is predicted considering patient's demographics, their living circumstances, comorbidities and symptoms [17]. More precisely, the following variables are used in our analysis:

Variable	Description	Data Type
Risk of infection	Risk of infection ≥ 12.56	Binary
Age	Patient’s age in years	Numerical
Gender	Patient’s gender	Categorical
Race	Patient’s race	Categorical
Smoking	Smoking status	Categorical
BMI	Body mass index	Numeric
House count	Number of household members	Numeric
Public transport count	Frequency of public transport	Numeric
Nursing home	Residency in a nursing home	Binary
COVID-19 symptoms	Occurrence of symptoms	Binary
COVID-19 contact	Contact to a COVID-19 positive person	Binary
Health worker	Working in healthcare	Binary
Asthma	Asthma	Binary
Kidney disease	Kidney disease	Binary
Liver disease	Liver disease	Binary
Heart disease	Heart disease	Binary
Lung disease	Lung disease	Binary
Diabetes	Diabetes mellitus	Binary
Hypertension	Hypertension	Binary

Table S9. Variables for the downstream analysis. The outcome variable is highlighted in italics. Data type gives information how the variable was measured.

Texas State Hospital Discharge Data

The Texas dataset contains the patient’s hospital discharge information for the first quarter of 2012 from Texas in the United States [18], and is publicly available at https://www.dshs.texas.gov/center-health-statistics/chs-data-sets-reports/texas-health-care-information-collection/health-data-researcher-information/texas-inpatient-public-use.

Using data from the Texas Inpatient Database, length of stay is predicted considering patient’s demographics, their health condition and charge information [19]. More precisely, the following variables are used in our analysis:

Variable	Description	Data Type
Length of stay	Length of stay ≥ 3 days	Binary
Age	Patient's age in years	Categorical
Gender	Patient's gender	Categorical
Race	Patient's race	Categorical
Hispanic ethnicity	Hispanic origin of patient	Binary
State	State of patient's mailing address in the USA	Categorical
Weekday of admission	Day of the week when patient was admitted (e.g. Monday)	Categorical
Risk mortality	Risk of mortality	Categorical
Disease severity	Severity of illness	Categorical
DRG	Diagnostic Related Group for prospective payment purposes	Categorical

Table S10. Variables for the downstream analysis. The outcome variable is highlighted in italics. Data type gives information how the variable was measured. DRG: Diagnostic Related Group.

Washington State Hospital Discharge Data 2007

The Washington dataset contains the patient's hospital 2007 discharge data from Washington, State Inpatient Databases (SID), Healthcare Cost and Utilization Project (HCUP), Agency for Healthcare Research and Quality [3], and is available for purchase at https://hcup-us.ahrq.gov/tech_assist/centdist.jsp.

Using data from the Washington Inpatient Database (2007), length of stay is predicted considering patient's demographics and their principal diagnosis [20], [21]. More precisely, the following variables are used in our analysis:

Variable	Description	Data Type
Length of stay	Length of stay ≥ 3 days	Binary
Age	Patient's age in years	Numeric
ZIP	Patient's zip code or living status (e.g. homeless)	Categorical
Admission type	Type of admission (e.g. emergency)	Categorical
Weekend admission	Admission on a weekend	Binary
DRG	Diagnostic Related Group for prospective payment purposes	Categorical
ICD-9	ICD-9 Diagnosis Code for principal diagnosis	Categorical
Deceased	Death of patient during inpatient stay	Binary

Table S11. Variables for the downstream analysis. The outcome variable is highlighted in italics. Data type gives information how the variable was measured. DRG: Diagnostic Related Group; ICD: International Classification of Disease.

Washington State Hospital Discharge Data 2008

The Washington2008 dataset contains the patient's hospital 2008 discharge data from Washington, State Inpatient Databases (SID), Healthcare Cost and Utilization Project (HCUP), Agency for Healthcare Research and Quality [3], and is available for purchase at https://hcup-us.ahrq.gov/tech_assist/centdist.jsp.

Using data from the Washington Inpatient Database (2008), length of stay is predicted considering patient's demographics, their principal diagnosis, comorbidities and undertaken procedures [21], [22]. More precisely, the following variables are used in our analysis:

Variable	Description	Data Type
Length of stay	Length of stay ≥ 3 days	Binary
Age	Patient's age in years	Numerical
Gender	Patient's gender	Binary
Race	Patient's race	Categorical
ZIP	Patient's zip code or living status (e.g. homeless)	Categorical
Admission type	Type of admission (e.g. emergency)	Categorical
Weekend admission	Admission on a weekend	Binary
DRG	Diagnostic Related Group for prospective payment purposes	Categorical
ICD-9	ICD-9 Diagnosis Code for principal diagnosis	Categorical
Chronic condition	Chronic condition of principal diagnosis	Binary
Body system	Body system affected by a chronic condition	Categorical
Procedure class	ICD Procedure Classes (e.g. minor diagnostic)	Categorical
Alcohol abuse	Alcohol abuse as comorbidity	Binary
Depression	Depressive disorder as comorbidity	Binary
Hypertension	Hypertension as comorbidity	Binary
Obesity	Obesity as comorbidity	Binary
Primary payer	Expected primary payer (e.g., Medicare)	Categorical

Table S12. Variables for the downstream analysis. The outcome variable is highlighted in italics. Data type gives information how the variable was measured. DRG: Diagnostic Related Group; ICD: International Classification of Disease.

Supplemental Methods

Sampling Scheme to Create Population Variants

We adopted a random weighted sampling scheme to reduce the number of population variants under investigation. This sampling process is described in more detail in the following.

We define v_0 as the number of variables in the *core* dataset, so the ones that are required for a pre-defined downstream modeling task. v are the number of *adjunct* variables that are in the dataset but not required for the downstream modeling task.

Then, the dimensionality of a dataset is defined by $v_0 + v$. The 12 reference populations had varying dimensionalities so that the maximum number of potential *adjunct* variables varied. This is referred to as pool size m. The larger the pool size, the higher the total number of potential combinations. We limited the maximum number of *adjunct* variables in this study to $k = \min(120, m)$ as datasets with a dimensionality of 120 already exhibited high complexity, and we did not expect additional insights form

including more variables. For two populations, namely Canadian COVID-19 (COVID-19) and Medical Information Mart for Intensive Care III (MIMIC-III), m was very small so that all possible combinations could be considered while the number of variants remained relatively small (31 and 15 respectively).

For all other healthcare populations, we considered a sample of all possible combinations. More precisely, for each v where v ranged from 1 to k, we examined r_v population variants. r_v was determined considering the entire space of potential population variants for that particular v:

$$r_v = \max\left(5,\ \left(w_v \times 600\right)\right) \tag{1}$$

With this formula, we ensured that for each v we considered at least 5 variants but also limited the maximum number of variants based on the weight w_v whereby 600 was a constant factor empirically chosen, so that, in total, the number of variants was between 600 and 750 per healthcare population. When the number of *adjunct* variables was equal to the pool size (i.e., $v = m$*),* then there was only one combination of the v *adjunct* variables, so that we set $r_v = 1$.

The weight w_v for each v was chosen to account for the different combinatory spaces across the healthcare populations and was calculated as:

$$w_v = \frac{\log\binom{m}{v}}{d} \tag{2}$$

with d acting as a normalizing factor considering all combinations among the potential *adjunct* variables:

$$d = \sum_{i=1}^{m} \log\binom{m}{i} \tag{3}$$

Discretization of Numerical Variables to Identify Hallucinations

To identify hallucinations, we applied row-wise anti-join between the synthetic data and the population variant (implemented via the dplyr R package [23]) which returns those records from the synthetic data that do not have an exact match in the corresponding population variant. Missing values were thereby handled as their own category.

To allow for some deviation from the real numerical value while still being considered as equal, we discretized numerical values as a pre-processing step. Discretizing means converting numerical values into categories. In this study, we discretized the value space of a numerical variable into 20 bins ranging from the minimum to the maximum value. This choice was informed by domain knowledge and the nature of our real-world data. Importantly, discretizing numerical values allowed for a more meaningful identification of hallucinations where divergences in categorical variables were treated as more relevant than those in numerical variables. This is clinically intuitive as differences in categorical variables such as a diagnosis of heart attack versus sepsis typically reflect fundamentally different conditions, while small numerical differences such as a patient aged 20 versus 21 would generally be considered as clinically negligible. Given that our reference populations included a limited set of numerical variables such as age, height, weight and BMI, we decided for a fixed bin number of 20 which results in clinically meaningful groupings (e.g. 5-year age intervals or 5 kg weight intervals).

While the bin number of 20 was informed by domain knowledge and the nature of our real-world data, it is important to acknowledge that the number of bins can influence the identification of hallucinations. To better understand the potential impact of discretization in this study, we report the proportion of discretized variables across all variants per population in the Supplemental Results (see section *Impact of Discretization on the HR*). The HR based on categorical variables only was calculated. In datasets where numerical variables contributed substantially to the HR (i.e., MIMIC-III and Nexoid), we additionally assessed the HR using an alternative discretizing approach with 40 bins ranging from the minimum to the maximum value. The rationale for increasing the number of bins is to align more closely with the conceptual definition of hallucinations as synthetic records that are non-existent in the population variant where the training data was sampled from. Such a more granular discretization is effectively less tolerant than the one in the main analysis. Results for this analysis are detailed in the Supplemental Results (see section *Impact of Discretization on the HR*).

Definition of Complexity

We considered dimensionality and cardinality in the definition of complexity. More precisely, for each population variant, the complexity was defined as the log-transformed combinations of the number of variables and their cardinality:

$$c = \log \sum_{i=1}^{v_0+v} u_i \tag{4}$$

where u_i was the cardinality of the variable i. For numerical variables, cardinality was set at their number of unique values, capped at 20 for numerical variables if the number exceeded that threshold. This threshold was consistent with the discretization of numerical variables when identifying hallucinations (see section *Discretization of Numerical Variables to Identify Hallucinations*).

This definition of complexity is a practical and interpretable way to describe structural complexity across datasets and intuitively captures the dataset characteristics that are most likely to influence hallucinations.

Synthetic Data Generation

7 different types of SDG models were considered when quantifying and analyzing hallucinations in SDG. In combination with the 6,354 population variants, this gives 44,478 trained SDG models each of which generated 10 synthetic datasets.

The included SDG models were sequential decision trees (ST) [24], Bayesian networks (BN) [25], conditional generative adversarial network (CTGAN) [26], variational autoencoders (TVAE and RTVAE) [26], adversarial random forests (ARF) [27], and normalizing flows (NFlow) [28]. Details are described in the following.

We used our implementation, the publicly available Python package pysdg [29], which builds upon the implementation described below with further pre- and post-processing.

Sequential Decision Trees

Similar to using a chaining method for multi-label classification problems, sequential decision trees (ST) generate synthetic data using conditional trees in a sequential fashion [24], [30], [31]. It has been commonly employed in the healthcare and social science domains for data synthesis [32], [33], [34], [35], [36], [37], [38], [39], [40]. The details of the implementation procedures can be referred to [24]. It was implemented using Aetion® Generate, a commercial product from Aetion[1].

Bayesian Networks

Bayesian Networks (BN) are models based on Directed Acyclic Graphs that consist of nodes representing the random variables and arcs representing the dependencies among these variables. To construct the BN model, the first step is to find the optimal network topology, and then to estimate the optimal parameters [41]. Starting with a random initial network structure, the Hill Climb heuristic search is used to find the optimal structure. Then, the conditional probability distributions are estimated using the maximum a posteriori estimator [42]. Once the network structure and the parameters are estimated, we can initialize the nodes with no incoming arcs by sampling from their marginal distributions and predict the rest of the connected variables using the estimated parameters. This was implemented using an open-sourced Python package Synthcity [43].

Conditional Generative Adversarial Network

A basic generative adversarial network (GAN) consists of two artificial neural networks (ANNs), a generator and a discriminator [44]. The generator and the discriminator play a min-max game. The input to the generator is noise, while its output is synthetic data. The discriminator has two inputs: the real training data and the synthetic data generated by the generator. The output of the discriminator indicates whether its input is real or synthetic. The generator is trained to 'trick' the discriminator by generating samples that look real. On the other hand, the discriminator is trained to maximize its discriminatory capability.

Among all the variations of GAN architectures, the conditional tabular GAN (CTGAN) is often used in tabular data synthesis [45]. CTGAN builds on conditional GANs by addressing the multimodal distributions of continuous variables and the highly imbalanced categorical variables [26]. CTGAN solves the first problem by proposing a per-mode normalization technique. For the second problem, each category of a categorical variable serves as the condition passed to the GAN. This was implemented using an open-sourced Python package Synthcity [43].

[1]See <https://aetion.com/products/generate/>

Variational Autoencoder

Variational autoencoders (VAE) use ANNs and involve two steps (encoding and decoding) to generate new samples [46]. First, an encoder is generated to compress input data into a lower-dimensional latent space, in which the data points are represented by distributions. The second step is a decoding process, in which new data samples are reconstructed as output from the latent space. The neural network is optimized by minimizing the reconstruction loss between the output and the input. VAEs are known to generate complex data of various types due to its ability to learn more complex distributions [47]. Many variants have been proposed as an extension of VAE, such as triplet-based VAE [48], conditional VAE [49], and Gaussian VAE [50]. In particular, the tabular VAE (TVAE) was proposed as an adaption of standard VAE to model and generate mixed-type tabular data with a modified loss function [26]. We also used a robust TVAE (RTVAE) as introduced by Akrami et al. [51]. This was implemented using an open-sourced Python package Synthcity [43].

Adversarial Random Forests (ARF)

Adversarial Random Forests is a tree-based density estimator that uses recursive unsupervised random forests [27]. This was implemented using an open-sourced Python package Synthcity [43].

Inspired by GANs, ARFs employ a recursive process where trees iteratively learn the structural properties of data by alternating between rounds of data generation and discrimination. This allows the model to gradually refine its understanding of the data distribution. Unlike classic tree-based models, ARFs provide smooth density estimations and can generate fully synthetic data.

Normalizing Flows (NFlow)

Normalizing Flows are generators based on monotonic rational-quadratic splines [28]. We used the implementation of NFlow from Synthcity [43]. NFlow utilizes monotonic rational-quadratic splines to implement invertible transformations, offering a significant improvement over traditional affine or additive transformations typically used in flow-based models.

Implementation of LGBM and MLP

Downstream task performance was assessed as Area under the Receiver Operating Characteristic curve (AUROC) and derived from two binary classifiers, namely LGBM and MLP.

Hyperparameters in LGBM were chosen in 5-fold-cross-validation via Bayesian Optimization [52]. We used the proposed ranges for the hyperparameters as suggested in [53], [54], [55], [56]. Cross-validation was also used to decide whether or not target encoding (in case of high cardinality variables), rebalancing via a sequential decision tree generative model [24], [57] and/or calibration via beta calibration [58], [59] was applied. We used the implementations in the sdgm R package [60]. Details with respect to the hyperparameters to select from are described in Table S13. If the algorithm failed, the hyperparameters were set to the default ones.

Hyperparameter	Default Value	Lower Bound	Upper Bound
Booster	1 (gradient boosting decision tree)	1 (gradient boosting decision tree)	2 (gradient-based one side sampling)
Maximal depth	6	1	15
Learning rate	log2(0.3)	-10	0
Early stopping rounds	7	7	30
Number of leaves	15	4	60
Minimal size of a leave	10	1	60

Table S13: Hyperparameter Range for LGBM.

In MLP, sequential classification model was built using the tensorflow and keras R package [61]. We used an input layer with 16 nodes, using Rectified Linear Unit (RLU) activation, a dropout layer with a dropout rate of 30% to prevent overfitting, a second hidden layer with 16 nodes, again using RLU activation, and an output layer with 1 node and a sigmoid activation function for binary classification. It was optimized with the Adam optimizer, binary cross-entropy as the loss function and accuracy as evaluation metric. Training was performed over 50 epochs. As mentioned in the main manuscript, extensive hyperparameter tuning was not conducted as exploratory results already demonstrated that this set-up yields comparable results to LGBM. We focused instead on avoiding overfitting [62] and internally split the data 80:20 to a training and validation dataset to monitor the model's performance on unseen synthetic data during training and to stop training as soon as the loss remained stable over two consecutive epochs.

Mixed-Effect Models

Mixed-effect models were used to assess the fixed component in our study while accounting for the population as a random effect. The random effect was very likely to not only affect the deviation from the mean but also the coefficient, so that it was included as random intercept and random slope. Generalized linear mixed-effect models using a binomial logit link function were chosen for the HR as outcome (in the sense of a series of binary trials, see details in [63]) and linear mixed-effect models for TSTR performance as outcome (see [64] for formula). Calculations were performed using the packages lme4 [64] and lmerTest [65] in R.

P values were computed for fixed effects via Satterthwaite approximation for degrees of freedom as described in [65]. The 95% confidence interval (CI) for the fixed effect estimates were calculated using the likelihood profile method. If the likelihood failed to converge such as when the model was too complex or data were sparse, we used the Wald CI as an approximation [64]. Model fit by marginal and conditional R^2 values was calculated by the package MuMIn [66].

Supplemental Results

Impact of Discretization on the HR

Hallucinations were defined as non-existent records in the corresponding population variant. This definition was operationalized by matching records between synthetic data and population variant and isolating those that were uniquely present in the synthetic data. To better understand the potential

impact of discretization in this approach, we conducted additional analyses as outlined in the Supplemental Methods.

Figure S1 shows the proportion of discretized variables across all variants per population. Most variants included predominantly categorical variables except for MIMIC-III which had a median proportion of discretized variables of 0.70 (IQR 0.67 - 0.73).

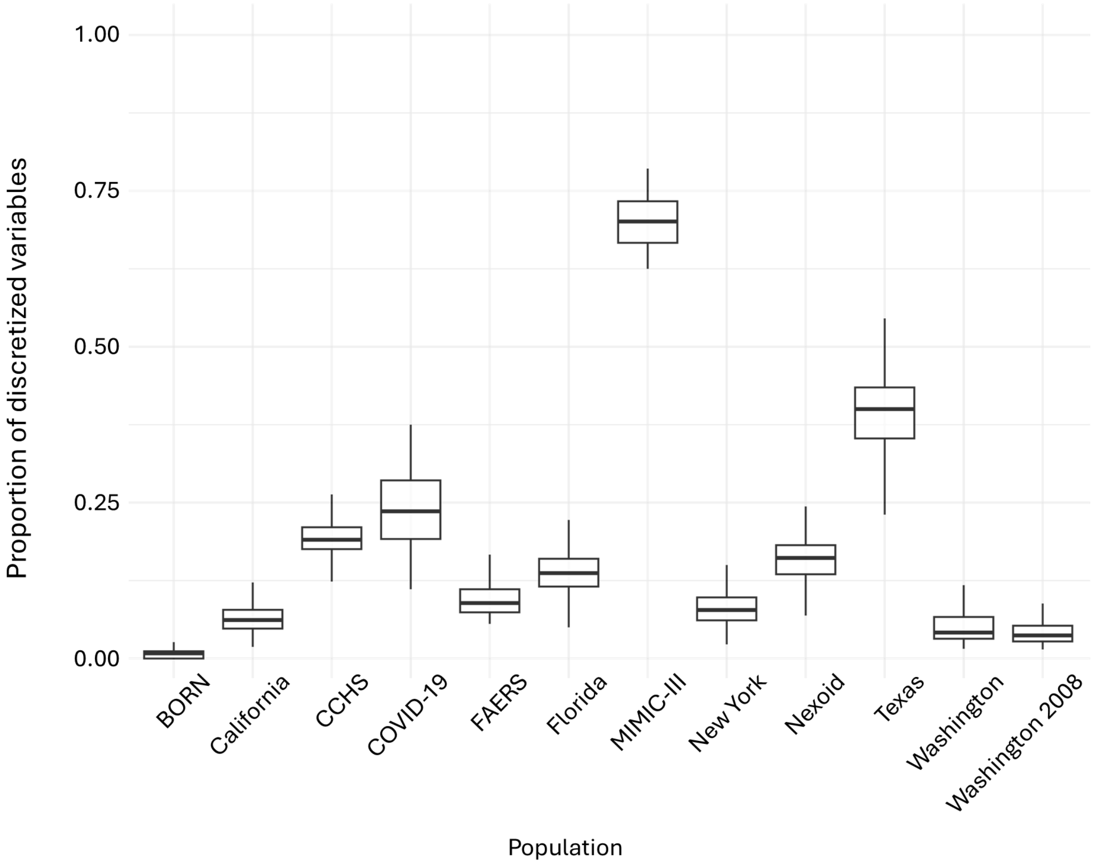

Figure S1: Proportion of discretized variables across all variants per Real-World Healthcare Population. To allow for some deviation from the real numerical value while still being considered as equal, we discretized numerical values as a pre-processing step. The proportion of variables affected by this pre-processing step are illustrated across all variants as a boxplot. The boxplots show the median as the central horizontal line, the lower and upper hinges represent the first and third quartiles (i.e., the interquartile range, IQR) and the whiskers represent the largest values within 1.5 times IQR from the quartiles.

The discretized variables contributed little to the HR, except for MIMIC-III and Nexoid, as shown by the calculation of the HR based on categorical variables only (see Table S14).

SDG Model	Population	HR			HR (Categorical Only)		
		**M**	**Q1**	**Q3**	**M**	**Q1**	**Q3**
ST	BORN	1.000	0.950	1.000	1.000	0.950	1.000
	California	0.980	0.965	0.995	0.978	0.960	0.994
	CCHS	1.000	1.000	1.000	1.000	1.000	1.000
	COVID-19	0.036	0.013	0.149	0.008	0.004	0.038
	FAERS	0.435	0.421	0.450	0.431	0.417	0.445
	Florida	0.999	0.984	1.000	0.998	0.980	1.000
	**MIMIC-III**	**0.975**	**0.964**	**0.979**	**0.008**	**0.004**	**0.011**
	New York	1.000	0.997	1.000	1.000	0.996	1.000
	**Nexoid**	**0.979**	**0.794**	**0.999**	**0.142**	**0.067**	**0.227**
	Texas	0.865	0.759	0.917	0.824	0.704	0.886
	Washington	0.963	0.928	0.991	0.957	0.912	0.990
	Washington 2008	0.987	0.958	1.000	0.983	0.952	0.999
BN	BORN	0.999	0.991	0.999	0.999	0.991	0.999
	California	0.994	0.985	0.999	0.993	0.984	0.999
	CCHS	0.886	0.868	0.994	0.568	0.554	0.911
	COVID-19	0.623	0.479	0.667	0.523	0.410	0.602
	FAERS	0.938	0.880	0.989	0.938	0.880	0.989
	Florida	0.998	0.995	1.000	0.998	0.994	0.999
	**MIMIC-III**	**0.862**	**0.858**	**0.880**	**0.117**	**0.082**	**0.177**
	New York	0.999	0.996	1.000	0.999	0.996	1.000
	Nexoid	0.994	0.894	1.000	0.272	0.122	0.561
	Texas	0.945	0.879	0.949	0.943	0.873	0.948
	Washington	0.998	0.993	0.999	0.998	0.992	0.999
	Washington 2008	0.999	0.997	0.999	0.999	0.997	0.999
ARF	BORN	1.000	0.988	1.000	1.000	0.988	1.000
	California	0.978	0.960	0.990	0.977	0.957	0.990
	CCHS	1.000	1.000	1.000	0.999	0.993	1.000
	COVID-19	0.369	0.174	0.409	0.219	0.094	0.357
	FAERS	0.980	0.964	0.988	0.980	0.964	0.988
	Florida	1.000	0.999	1.000	1.000	0.999	1.000
	**MIMIC-III**	**0.993**	**0.988**	**0.995**	**0.234**	**0.163**	**0.312**
	New York	1.000	1.000	1.000	1.000	1.000	1.000
	**Nexoid**	**0.988**	**0.873**	**0.999**	**0.274**	**0.112**	**0.577**
	Texas	0.948	0.911	0.974	0.925	0.879	0.950
	Washington	0.998	0.994	1.000	0.998	0.994	1.000
	Washington 2008	0.999	0.995	1.000	0.999	0.995	1.000
CTGAN	BORN	1.000	0.982	1.000	1.000	0.982	1.000
	California	1.000	1.000	1.000	1.000	1.000	1.000
	CCHS	1.000	1.000	1.000	1.000	0.993	1.000
	COVID-19	0.883	0.813	0.949	0.796	0.662	0.902
	FAERS	0.980	0.911	0.996	0.980	0.911	0.996
	Florida	1.000	1.000	1.000	1.000	1.000	1.000
	**MIMIC-III**	**0.992**	**0.986**	**0.994**	**0.135**	**0.083**	**0.213**
	New York	1.000	1.000	1.000	1.000	1.000	1.000
	**Nexoid**	**0.986**	**0.859**	**0.999**	**0.277**	**0.125**	**0.572**

	Texas	1.000	0.997	1.000	1.000	0.996	1.000
	Washington	1.000	1.000	1.000	1.000	1.000	1.000
	Washington 2008	1.000	1.000	1.000	1.000	1.000	1.000
TVAE	BORN	1.000	0.962	1.000	1.000	0.962	1.000
	California	1.000	1.000	1.000	1.000	1.000	1.000
	CCHS	1.000	1.000	1.000	0.999	0.984	1.000
	COVID-19	0.837	0.728	0.919	0.732	0.521	0.876
	FAERS	0.975	0.888	0.990	0.974	0.888	0.990
	Florida	1.000	1.000	1.000	1.000	1.000	1.000
	**MIMIC-III**	**0.981**	**0.971**	**0.986**	**0.102**	**0.034**	**0.132**
	New York	1.000	1.000	1.000	1.000	1.000	1.000
	**Nexoid**	**0.969**	**0.792**	**0.998**	**0.156**	**0.055**	**0.483**
	Texas	0.999	0.994	1.000	0.999	0.992	1.000
	Washington	1.000	1.000	1.000	1.000	1.000	1.000
	Washington 2008	1.000	1.000	1.000	1.000	1.000	1.000
RTVAE	BORN	1.000	0.957	1.000	1.000	0.957	1.000
	California	1.000	1.000	1.000	1.000	1.000	1.000
	CCHS	1.000	1.000	1.000	0.999	0.984	1.000
	COVID-19	0.926	0.782	0.966	0.839	0.665	0.943
	FAERS	1.000	0.899	1.000	1.000	0.899	1.000
	Florida	1.000	1.000	1.000	1.000	1.000	1.000
	**MIMIC-III**	**0.998**	**0.996**	**0.998**	**0.131**	**0.073**	**0.275**
	New York	1.000	1.000	1.000	1.000	1.000	1.000
	**Nexoid**	**0.994**	**0.835**	**1.000**	**0.147**	**0.049**	**0.758**
	Texas	1.000	1.000	1.000	1.000	1.000	1.000
	Washington	1.000	1.000	1.000	1.000	1.000	1.000
	Washington 2008	1.000	1.000	1.000	1.000	1.000	1.000
NFlow	BORN	1.000	0.999	1.000	1.000	0.999	1.000
	California	1.000	1.000	1.000	1.000	1.000	1.000
	CCHS	1.000	1.000	1.000	1.000	1.000	1.000
	COVID-19	0.926	0.817	0.969	0.837	0.738	0.938
	FAERS	0.992	0.967	0.998	0.992	0.966	0.998
	Florida	1.000	1.000	1.000	1.000	1.000	1.000
	**MIMIC-III**	**0.997**	**0.995**	**0.999**	**0.167**	**0.119**	**0.229**
	New York	1.000	1.000	1.000	1.000	1.000	1.000
	**Nexoid**	**0.999**	**0.962**	**1.000**	**0.537**	**0.302**	**0.733**
	Texas	1.000	1.000	1.000	1.000	0.999	1.000
	Washington	1.000	1.000	1.000	1.000	1.000	1.000
	Washington 2008	1.000	1.000	1.000	1.000	1.000	1.000

Table S14: Hallucination Rate (HR) for Categorical Variables per SDG Model and Population. The HR was calculated as described in the main manuscript. Additionally, the HR was calculated using categorical variables only to better understand the potential impact of discretizing numerical variables. The HR is summarized across all population variants of the respective population. Populations with relevant proportions of numerical variables so that the HR deviate are printed in bold. M: median; Q1: first quartile (25th percentile); Q3: third quartile (75th percentile).

To better understand the potential contribution of the chosen number of bins in the affected populations, namely Nexoid and MIMIC-III, we calculated the HR using an alternative discretizing approach with 40 bins ranging from the minimum to the maximum value. This is shown in Figure S2 and Figure S3. As expected, the more granular discretization resulted in a higher HR; the median values, however, remained largely comparable across the SDG models.

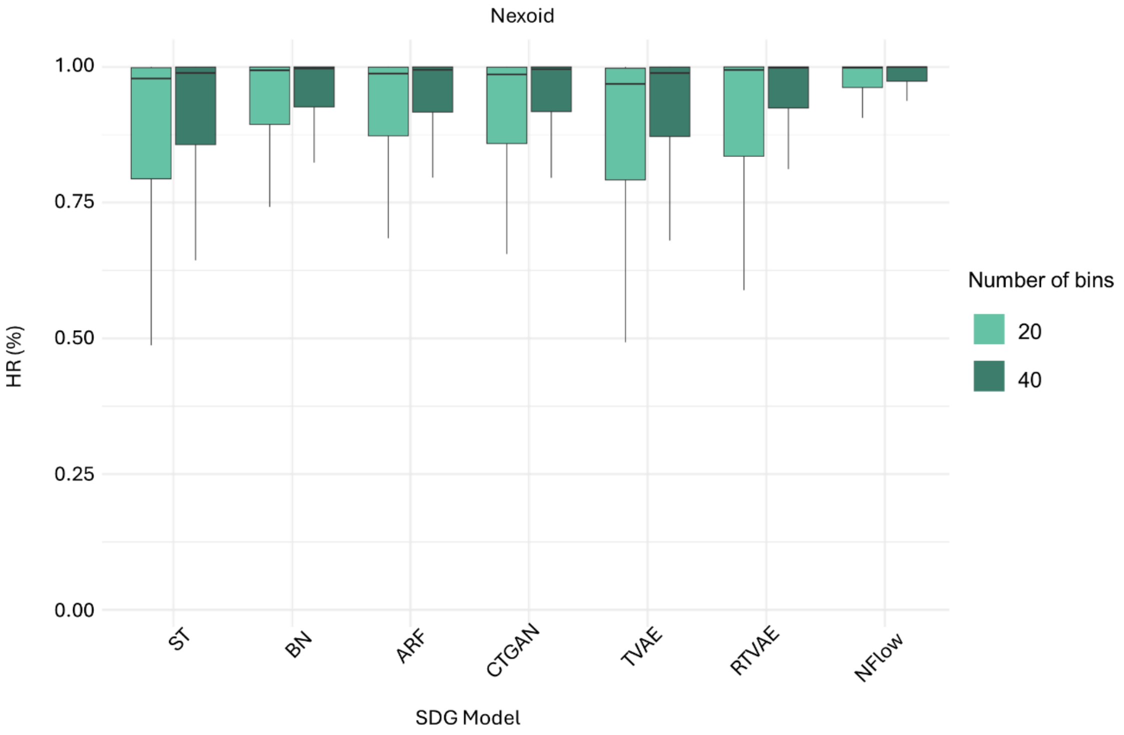

Figure S2: Hallucination Rate (HR) per SDG Model in Nexoid With Different Number of Bins. The HR was calculated as described in the main manuscript and numerical variables were discretized into 20 bins ranging from the minimum to the maximum value. Additionally, the HR was calculated when numerical variables were discretized into 40 bins ranging from the minimum to the maximum value. The HR across the population variants of Nexoid are illustrated as a boxplot for each SDG model. The boxplots show the median as the central horizontal line, the lower and upper hinges represent the first and third quartiles (i.e., the interquartile range, IQR) and the whiskers represent the largest values within 1.5 times IQR from the quartiles.

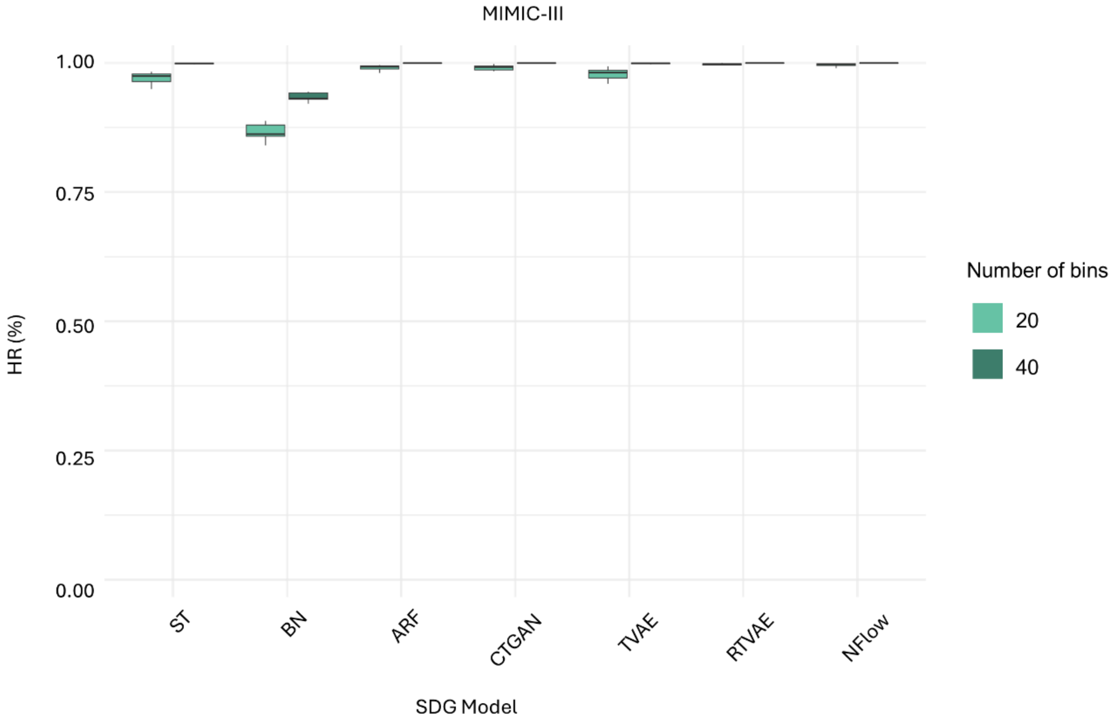

Figure S3: Hallucination Rate (HR) per SDG Model in MIMIC-III With Different Number of Bins. The HR was calculated as described in the main manuscript and numerical variables were discretized into 20 bins ranging from the minimum to the maximum value. Additionally, the HR was calculated when numerical variables were discretized into 40 bins ranging from the minimum to the maximum value. The HR across the population variants of Nexoid are illustrated as a boxplot for each SDG model. The boxplots show the median as the central horizontal line, the lower and upper hinges represent the first and third quartiles (i.e., the interquartile range, IQR) and the whiskers represent the largest values within 1.5 times IQR from the quartiles.

Sensitivity Analysis: Effect of Complexity on HR with 50% and 25% of the Population Variants

Given the large scale of our experiments, an important question is whether a smaller number of population variants would give the same results for the effect of complexity on HR. We conducted a sensitivity analysis by randomly selecting 50% and 25% of the population variants per population and estimating the effect of complexity on the HR analogous to the main analysis. The 50% subset could confirm a significant fixed effect of complexity on the HR with almost identical effect sizes (see Table S15).

SDG model	Fixed effect complexity (OR)	95% CI (lower)	95% CI (upper)	p-value	R^2 (fixed effect)	R^2 (overall)
ST	1.07	1.03	1.11	0.0005	0.25	0.99
BN	1.03	1.01	1.05	0.0075	0.14	0.99
ARF	1.07	1.03	1.12	0.0002	0.31	0.99
CTGAN	1.11	1.06	1.15	< 0.0001	0.40	0.99
TVAE	1.11	1.07	1.15	< 0.0001	0.46	0.99
RTVAE	1.14	1.08	1.20	< 0.0001	0.39	0.99
NFlow	1.16	1.11	1.21	< 0.0001	0.46	0.99

Table S15: Modeling the Effect of Complexity on the HR with 50% of the Population Variants. Generalized linear mixed effect models were fitted for each SDG model separately with a random 50% subset of all population variants per population: 3,180 for ST, 3,180 for BN, 3,180 for ARF, 3,180 for CTGAN, 3,180 for TVAE, 3,179 for RTVAE and 3,168 for NFlow. The population was considered as random effect, complexity as fixed effect and the HR as outcome. The odds for hallucinations (OR) are indicated. We provide the variance explained (i.e., R^2) by the fixed effect only and by both fixed and marginal effects together (i.e., R^2 overall) for all models.

The fixed effect of complexity on the HR could be replicated with even fewer population variants, more precisely with a 25% subset of all population variants per population (see Table S16).

SDG model	Fixed effect complexity (OR)	95% CI (lower)	95% CI (upper)	p-value	R^2 (fixed effect)	R^2 (overall)
ST	1.08	1.03	1.13	0.0002	0.23	0.99
BN	1.03	1.00	1.07	0.0040	0.19	0.99
ARF	1.07	1.03	1.12	0.0001	0.25	0.99
CTGAN	1.13	1.08	1.17	< 0.0001	0.52	0.99
TVAE	1.11	1.06	1.15	< 0.0001	0.43	0.99
RTVAE	1.18	1.10	1.27	< 0.0001	0.43	0.99
NFlow	1.16	1.11	1.22	< 0.0001	0.55	0.99

Table S16: Modeling the Effect of Complexity on the HR with 25% of the Population Variants. Generalized linear mixed effect models were fitted for each SDG model separately with a random 25% subset of all population variants per population: 1,594 for ST, 1,594 for BN, 1,594 for ARF, 1,594 for CTGAN, 1,594 for TVAE, 1,593 for RTVAE and 1,585 for NFlow. The population was considered as random effect, complexity as fixed effect and the HR as outcome. The odds for hallucinations (OR) are indicated. We provide the variance explained (i.e., R^2) by the fixed effect only and by both fixed and marginal effects together (i.e., R^2 overall) for all models.

Sensitivity Analysis: Effect of HR on TSTR with 50% and 25% of the Population Variants

The same considerations applied to the effect of HR on TSTR. To better understand whether a smaller number of population variants would give the same results for the effect of HR on TSTR, we conducted another sensitivity analysis with the same randomly selected 50% and 25% of the population variants per population by estimating the effect of the HR on downstream prognostic AI/ML modeling performance (i.e., TSTR) analogous to the main analysis.

The prognostic AI/ML performance across all downstream tasks was similar to the main analysis in both cases, the 25% and 50% subset (see Table S17 and Table S18).

Population	LGBM									MLP								
	TRTR			TSTR			TRTR-TSTR			TRTR			TSTR			TRTR-TSTR		
	M	Q1	Q3	M	Q1	Q3	M	Q1	Q3	M	Q1	Q3	M	Q1	Q3	M	Q1	Q3
BORN	0.923	0.922	0.924	0.907	0.898	0.911	0.017	0.011	0.025	0.896	0.896	0.896	0.864	0.850	0.877	0.032	0.019	0.046
California	0.810	0.808	0.812	0.665	0.633	0.721	0.144	0.089	0.176	0.854	0.854	0.854	0.824	0.804	0.839	0.030	0.015	0.050
CCHS	0.708	0.706	0.709	0.664	0.639	0.683	0.043	0.026	0.068	0.698	0.698	0.698	0.694	0.688	0.698	0.004	0.000	0.010
COVID-19	0.958	0.954	0.959	0.768	0.628	0.914	0.190	0.045	0.328	0.931	0.931	0.931	0.742	0.664	0.827	0.190	0.105	0.268
FAERS	0.663	0.653	0.675	0.557	0.538	0.574	0.105	0.086	0.127	0.928	0.928	0.928	0.816	0.771	0.862	0.111	0.065	0.157
Florida	0.750	0.747	0.751	0.622	0.596	0.644	0.128	0.106	0.154	0.837	0.837	0.837	0.811	0.789	0.826	0.026	0.011	0.048
Mimic-III	0.655	0.652	0.657	0.562	0.550	0.574	0.093	0.079	0.106	0.534	0.534	0.534	0.527	0.522	0.532	0.007	0.002	0.012
New York	0.806	0.801	0.806	0.651	0.626	0.685	0.153	0.118	0.178	0.859	0.859	0.859	0.832	0.810	0.848	0.028	0.011	0.049
Nexoid	0.731	0.729	0.731	0.676	0.662	0.701	0.054	0.029	0.068	0.681	0.681	0.681	0.683	0.671	0.692	-0.002	-0.011	0.010
Texas	0.810	0.808	0.810	0.747	0.720	0.762	0.062	0.048	0.090	0.813	0.813	0.813	0.787	0.778	0.800	0.025	0.012	0.035
Washington	0.784	0.782	0.788	0.651	0.617	0.679	0.135	0.106	0.168	0.870	0.870	0.870	0.844	0.831	0.852	0.026	0.017	0.038
Washington 2008	0.808	0.806	0.812	0.683	0.648	0.709	0.126	0.099	0.160	0.877	0.877	0.877	0.843	0.827	0.858	0.033	0.019	0.049

Table S17: Downstream Prognostic AI/ML Modeling Performance with 50% of the Population Variants. The different downstream tasks achieved varying performance in the real data (TRTR). The performance achieved by training on the random 50% subset of all population variants is indicated as TSTR and summarized across all SDG models. The deviation of the performance derived from the synthetic data (TSTR) is indicated as TRTR-TSTR. Performance was measured as AUROC. M: median; Q1: first quartile (25th percentile); Q3: third quartile (75th percentile).

Population	LGBM									MLP								
	TRTR			TSTR			TRTR-TSTR			TRTR			TSTR			TRTR-TSTR		
	M	Q1	Q3	M	Q1	Q3	M	Q1	Q3	M	Q1	Q3	M	Q1	Q3	M	Q1	Q3
BORN	0.923	0.922	0.924	0.906	0.898	0.911	0.017	0.012	0.025	0.896	0.896	0.896	0.864	0.850	0.877	0.032	0.019	0.046
California	0.810	0.808	0.812	0.666	0.632	0.722	0.145	0.088	0.178	0.854	0.854	0.854	0.823	0.805	0.838	0.031	0.016	0.049
CCHS	0.708	0.706	0.710	0.665	0.640	0.683	0.043	0.025	0.067	0.698	0.698	0.698	0.694	0.688	0.698	0.004	-0.001	0.009
COVID-19	0.957	0.955	0.958	0.796	0.639	0.929	0.162	0.030	0.318	0.931	0.931	0.931	0.737	0.658	0.822	0.194	0.109	0.273
FAERS	0.662	0.651	0.676	0.557	0.538	0.575	0.105	0.086	0.127	0.928	0.928	0.928	0.816	0.767	0.864	0.112	0.063	0.161
Florida	0.750	0.747	0.751	0.622	0.597	0.643	0.128	0.106	0.152	0.837	0.837	0.837	0.811	0.787	0.824	0.026	0.012	0.050
Mimic-III	0.659	0.650	0.663	0.559	0.551	0.575	0.096	0.074	0.107	0.534	0.534	0.534	0.531	0.525	0.535	0.004	0.000	0.010
New York	0.806	0.801	0.806	0.651	0.627	0.685	0.153	0.118	0.177	0.859	0.859	0.859	0.832	0.810	0.848	0.028	0.011	0.050
Nexoid	0.731	0.729	0.731	0.676	0.662	0.702	0.054	0.028	0.068	0.681	0.681	0.681	0.684	0.672	0.693	-0.003	-0.012	0.009
Texas	0.810	0.808	0.811	0.747	0.718	0.762	0.062	0.048	0.091	0.813	0.813	0.813	0.787	0.778	0.800	0.026	0.012	0.035
Washington	0.784	0.782	0.788	0.650	0.617	0.679	0.135	0.105	0.167	0.870	0.870	0.870	0.844	0.832	0.852	0.025	0.017	0.038
Washington 2008	0.808	0.806	0.809	0.683	0.649	0.708	0.126	0.100	0.159	0.877	0.877	0.877	0.843	0.827	0.857	0.034	0.020	0.050

Table S18: Downstream Prognostic AI/ML Modeling Performance with 25% of the Population Variants. The different downstream tasks achieved varying performance in the real data (TRTR). The performance achieved by training on the random 25% subset of all population variants is indicated as TSTR and summarized across all SDG models. The deviation of the performance derived from the synthetic data (TSTR) is indicated as TRTR-TSTR. Performance was measured as AUROC. M: median; Q1: first quartile (25th percentile); Q3: third quartile (75th percentile).

The downstream performance was modeled using the HR as fixed effect and the population as random effect, analogous to the main analysis but with the 25% and 50% subset of all population variants. In contrast to the previous sensitivity analysis, differences in statistical significance could be observed when modeling the influence of the HR as a fixed effect on the downstream prognostic AI/ML modeling performance (see Table S19 and Table S20). These were more pronounced with the 25% subset. While these differences would not alter the main conclusion, namely that there was no relevant negative or positive trend in the effect of HR on prognostic AI/ML model performance, the reduction of population variants led to a reduction in the number of populations where the HR was sufficiently spread so that they were included as random effect. In the 50% subset, the filtering step reduced the subset for effect modeling to 19.7% (4,383/22,260 trained SDG models) but for each SDG model the same populations were included as random effect. In the 25% subset, the filtering step reduced the subset for effect modeling to 14.8% (1,654/11,158 trained SDG models) which resulted in a sparse coverage where some SDG models had no random effect or a random effect with only two populations to account for.

This means that fewer population variants gave similar effect estimates but reliability was lower due to the insufficient spread of the HR across these variants.

SDG model	AI/ML model	Fixed effect HR (OR)	95% CI (lower)	95% CI (upper)	p-value	R^2 (fixed effect)	R^2 (overall)
ST	LGBM	0.0000	-0.0001	0.0002	0.5967	0.0000	0.9958
	MLP	-0.0001	-0.0001	0.0000	0.1948	0.0004	0.9905
BN	LGBM	0.0000	-0.0001	0.0001	0.9053	0.0000	0.9930
	MLP	0.0003	0.0001	0.0006	0.1071	0.0031	0.9674
ARF	LGBM	-0.0002	-0.0004	0.0000	0.2589	0.0009	0.9984
	MLP	-0.0001	-0.0002	-0.0001	0.0106	0.0009	0.9915
CTGAN	**LGBM**	**-0.0002**	**-0.0003**	**-0.0001**	**< 0.0001**	**0.0001**	**0.9892**
	MLP	-0.0002	-0.0003	-0.0001	0.1014	0.0007	0.9838
TVAE	LGBM	0.0003	-0.0002	0.0008	0.4339	0.0025	0.9717
	MLP	0.0000	-0.0001	0.0001	0.5712	0.0000	0.9725
RTVAE	LGBM	0.0000	-0.0002	0.0001	0.8646	0.0000	0.9707
	MLP	-0.0001	-0.0001	0.0000	0.0958	0.0003	0.9749
NFlow	LGBM	-0.0007	-0.0039	0.0024	0.6303	0.1161	0.1161
	MLP	0.0006	-0.0022	0.0034	0.6530	0.0001	0.0001

Table S19: Modeling the Effect of HR on the Downstream Performance with 50% of the Population Variants. Linear mixed effect models were fitted for each SDG model separately with a random 50% subset of all population variants per population: 980 (LGBM) and 982 (MLP) for ST, 677 (LGBM and MLP) for BN, 677 (LGBM and MLP) for ARF, 677 (LGBM and MLP) for CTGAN, 677 (LGBM and MLP) for TVAE, 675 (LGBM) and 677 (MLP) for RTVAE and 16 (LGBM and MLP) for NFlow. Populations were considered as random effect, HR as fixed effect and the TSTR as outcome. Both, LGBM and MLP, are considered. The coefficients for the HR in [%] are indicated. Models with a p-value < 0.05 are highlighted in bold. We provide the variance explained (i.e., R^2) by the fixed effect only and by both fixed and marginal effects together (i.e., R^2 overall) for all models. Note that for NFlow, there was no random effect since only one population met the requirements of HR range so that R^2 and R^2 overall are identical.

	AI/ML model	Fixed effect HR (OR)	95% CI (lower)	95% CI (upper)	p-value	R^2 (fixed effect)	R^2 (overall)
ST	LGBM	0.0000	-0.0001	0.0002	0.6443	0.0000	0.9958
	MLP	-0.0001	-0.0001	0.0000	0.2262	0.0002	0.9915
BN	LGBM	0.0003	-0.0005	0.0010	0.7046	0.0052	0.9274
	MLP	0.0006	-0.0006	0.0017	0.5809	0.0133	0.9559
ARF	LGBM	-0.0002	-0.0002	-0.0001	0.2847	0.0005	0.9987
	MLP	-0.0002	-0.0003	0.0000	0.5611	0.0018	0.9855
CTGAN	**LGBM**	**-0.0002**	**-0.0003**	**-0.0001**	**< 0.0001**	**0.1313**	**0.1313**
	**MLP**	**-0.0002**	**-0.0002**	**-0.0001**	**< 0.0001**	**0.1281**	**0.1281**
TVAE	LGBM	0.0010	-0.0009	0.0029	0.4253	0.0289	0.9800
	MLP	0.0005	-0.0006	0.0016	0.4591	0.0109	0.9841
RTVAE	LGBM	0.0000	-0.0003	0.0003	0.9497	0.0000	0.9927
	MLP	-0.0001	-0.0002	0.0000	0.0121	0.0003	0.9866
NFlow	LGBM	-0.0012	-0.0073	0.0048	0.6393	0.0336	0.0336
	MLP	0.0031	-0.0022	0.0084	0.2028	0.2260	0.2260

Table S20: Modeling the Effect of HR on the Downstream Performance with 25% of the Population Variants. Linear mixed effect models were fitted for each SDG model separately with a random 25% subset of all population variants per population: 491 (LGBM) and 492 (MLP) for ST, 164 (LGBM and MLP) for BN, 164 (LGBM and MLP) for ARF, 156 (LGBM and MLP) for CTGAN, 337 (LGBM) and 339 (MLP) for TVAE, 331 (LGBM and MLP) for RTVAE and 8 (LGBM and MLP) for NFlow. Populations were considered as random effect, HR as fixed effect and the TSTR as outcome. Both, LGBM and MLP, are considered. The coefficients for the HR in [%] are indicated. Models with a p-value < 0.05 are highlighted in bold. We provide the variance explained (i.e., R^2) by the fixed effect only and by both fixed and marginal effects together (i.e., R^2 overall) for all models. Note that for NFlow and CTGAN, there were no random effects since only one population met the requirements of HR range so that R^2 and R^2 overall are identical.

Robustness of TRTR Over 10 Different Splits

Since splitting a dataset into training and holdout sets introduces stochasticity, we repeated the evaluation over 10 different training-holdout splits to ensure robustness of the downstream performance results to the split. TRTR was measured for LGBM and MLP as described in the main manuscript. There was very little variation in the AUROC for LGBM across the 10 splits (see Figure S4). For MLP, this was also true for most populations. The population FAERS, however, had a larger IQR of 0.667-0.911 (median 0.862). The split in the main analysis yielded an AUROC of 0.928, thereby being above the third quartile.

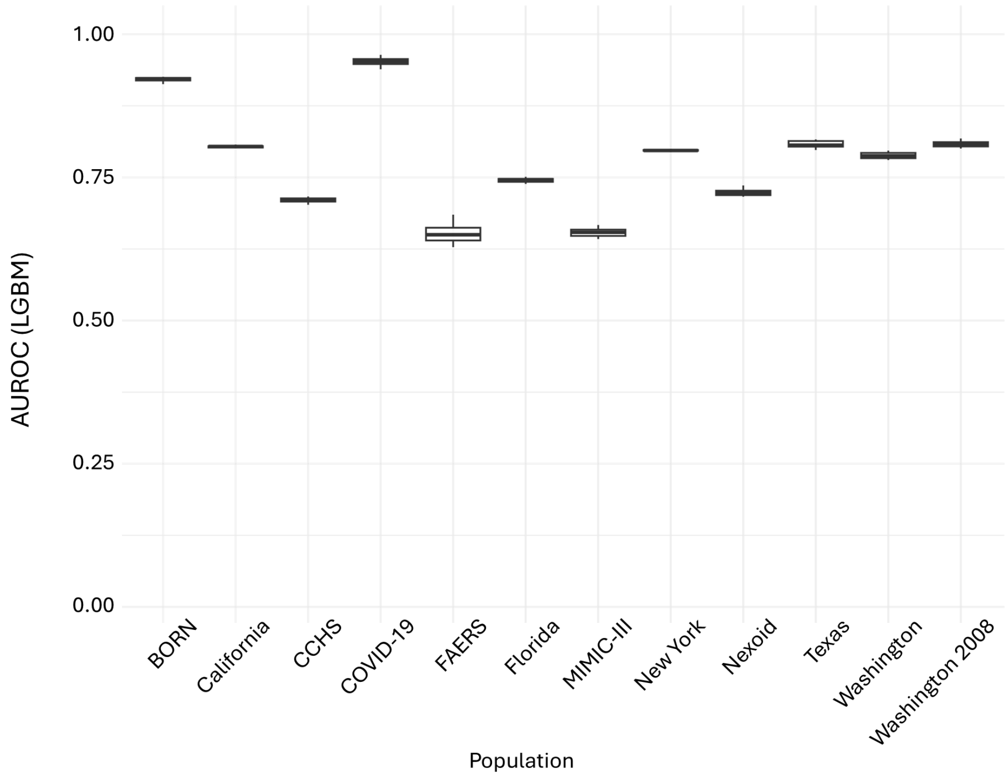

Figure S4: Downstream Prognostic AI/ML Modeling Performance for LGBM Across 10 Different Training-Holdout Splits. The performance in the real data (TRTR) was calculated over 10 additional splits to investigate whether results from our main analysis are sensitive to the split. Performance was measured as AUROC. The boxplots show the median as the central horizontal line, the lower and upper hinges represent the first and third quartiles (i.e., the interquartile range, IQR) and the whiskers represent the largest values within 1.5 times IQR from the quartiles.

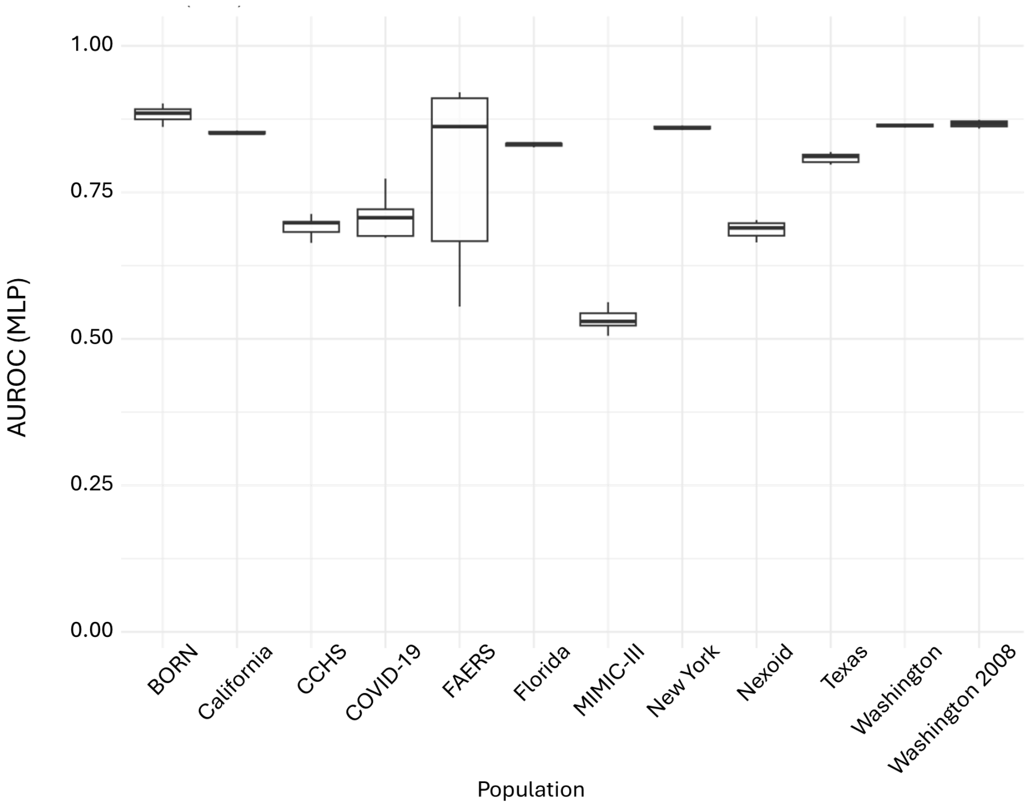

Figure S5: Downstream Prognostic AI/ML Modeling Performance for MLP Across 10 Different Training-Holdout Splits. The performance in the real data (TRTR) was calculated over 10 additional splits to investigate whether results from our main analysis are sensitive to the split. Performance was measured as AUROC. The boxplots show the median as the central horizontal line, the lower and upper hinges represent the first and third quartiles (i.e., the interquartile range, IQR) and the whiskers represent the largest values within 1.5 times IQR from the quartiles.

References

[1] J. Valero De Bernabé *et al.*, "Risk factors for low birth weight: a review," *Eur J Obstet Gynecol Reprod Biol*, vol. 116, no. 1, pp. 3–15, Sep. 2004, doi: 10.1016/j.ejogrb.2004.03.007.

[2] D. K. Yadav, U. Chaudhary, and N. Shrestha, "Risk factors associated with low birth weight," *J Nepal Health Res Counc*, vol. 9, no. 2, pp. 159–164, Oct. 2011.

[3] "HCUP State Inpatient Databases (SID). Healthcare Cost and Utilization Project (HCUP). 2005-2009. Agency for Healthcare Research and Quality, Rockville, MD. www.hcup-us.ahrq.gov/sidoverview.jsp."

[4] F. Ul and M. Ml, "Frequency, trends, and antecedents of severe maternal depression after three million U.S. births," *PloS one*, vol. 13, no. 2, Feb. 2018, doi: 10.1371/journal.pone.0192854.

[5] S. A. Brownlee *et al.*, "Impact of Post-Hospital Syndrome on Outcomes Following Elective, Ambulatory Surgery," *Ann Surg*, vol. 266, no. 2, pp. 274–279, Aug. 2017, doi: 10.1097/SLA.0000000000001965.

[6] L. C. Maclagan *et al.*, “The CANHEART health index: a tool for monitoring the cardiovascular health of the Canadian population,” *CMAJ*, vol. 186, no. 3, pp. 180–187, Feb. 2014, doi: 10.1503/cmaj.131358.
[7] I. Berry *et al.*, “A sub-national real-time epidemiological and vaccination database for the COVID-19 pandemic in Canada,” *Sci Data*, vol. 8, no. 1, p. 173, Jul. 2021, doi: 10.1038/s41597-021-00955-2.
[8] K. Marwitz, S. C. Jones, C. M. Kortepeter, G. J. Dal Pan, and M. A. Muñoz, “An Evaluation of Postmarketing Reports with an Outcome of Death in the US FDA Adverse Event Reporting System,” *Drug Saf*, vol. 43, no. 5, pp. 457–465, May 2020, doi: 10.1007/s40264-020-00908-5.
[9] J. Meddings *et al.*, “The Impact of Disability and Social Determinants of Health on Condition-Specific Readmissions beyond Medicare Risk Adjustments: A Cohort Study,” *J Gen Intern Med*, vol. 32, no. 1, pp. 71–80, Jan. 2017, doi: 10.1007/s11606-016-3869-x.
[10] A. Johnson, T. Pollard, and R. Mark, “MIMIC-III Clinical Database (version 1.4).” PhysioNet, PhysioNet, PhysioNet 2016. doi: https://doi.org/10.13026/C2XW26.
[11] A. E. W. Johnson *et al.*, “MIMIC-III, a freely accessible critical care database,” *Sci Data*, vol. 3, no. 1, Art. no. 1, May 2016, doi: 10.1038/sdata.2016.35.
[12] A. L. Goldberger *et al.*, “PhysioBank, PhysioToolkit, and PhysioNet: Components of a New Research Resource for Complex Physiologic Signals,” *Circulation*, vol. 101, no. 23, Jun. 2000, doi: 10.1161/01.CIR.101.23.e215.
[13] M. Pishgar, J. Theis, M. Del Rios, A. Ardati, H. Anahideh, and H. Darabi, “Prediction of unplanned 30-day readmission for ICU patients with heart failure,” *BMC Med Inform Decis Mak*, vol. 22, no. 1, p. 117, May 2022, doi: 10.1186/s12911-022-01857-y.
[14] O. Aliu *et al.*, “The effect of pre-Affordable Care Act (ACA) Medicaid eligibility expansion in New York State on access to specialty surgical care,” *Med Care*, vol. 52, no. 9, pp. 790–795, Sep. 2014, doi: 10.1097/MLR.0000000000000175.
[15] J. M. Kahn *et al.*, “The Epidemiology of Chronic Critical Illness in the United States,” *Crit Care Med*, vol. 43, no. 2, pp. 282–287, Feb. 2015, doi: 10.1097/CCM.0000000000000710.
[16] A. K. Sabbatini, K. E. Kocher, A. Basu, and R. Y. Hsia, “In-Hospital Outcomes and Costs Among Patients Hospitalized During a Return Visit to the Emergency Department,” *JAMA*, vol. 315, no. 7, pp. 663–671, Feb. 2016, doi: 10.1001/jama.2016.0649.
[17] J. Grantham, “COVID-19 Survival Calculator,” Nexoid’s COVID-19 Survival Calculator. Accessed: Dec. 22, 2020. [Online]. Available: https://www.covid19survivalcalculator.com
[18] “Texas Hospital Inpatient Discharge Public Use Data File, First Quarter, 2012, 1st quarter 2012. Texas Department of State Health Services, Center for Health Statistics, Austin, Texas.”
[19] J. Zhang and P. Yu, “Machine Learning Methods for Prediction of COVID-19 Patient Length of Stay: Using Texas PUDF Data,” in *2023 3rd International Conference on Electrical, Computer, Communications and Mechatronics Engineering (ICECCME)*, Jul. 2023, pp. 1–7. doi: 10.1109/ICECCME57830.2023.10252792.
[20] L. B. Goss, J. R. Ortiz, D. M. Okamura, K. Hayward, and C. H. Goss, “Significant Reductions in Mortality in Hospitalized Patients with Systemic Lupus Erythematosus in Washington State from 2003 to 2011,” *PLoS One*, vol. 10, no. 6, p. e0128920, 2015, doi: 10.1371/journal.pone.0128920.
[21] D. Metcalfe, C. K. Zogg, E. R. Haut, T. M. Pawlik, A. H. Haider, and D. C. Perry, “Data resource profile: State Inpatient Databases,” *International Journal of Epidemiology*, vol. 48, no. 6, pp. 1742–1742h, Dec. 2019, doi: 10.1093/ije/dyz117.
[22] M. L. Barrett, L. M. Wier, H. J. Jiang, and C. A. Steiner, “All-Cause Readmissions by Payer and Age, 2009–2013,” in *Healthcare Cost and Utilization Project (HCUP) Statistical Briefs [Internet]*, Agency for Healthcare Research and Quality (US), 2015. Accessed: Oct. 14, 2024. [Online]. Available: https://www.ncbi.nlm.nih.gov/books/NBK343800/

[23] H. Wickham *et al.*, *dplyr: A Grammar of Data Manipulation*. (Nov. 17, 2023). Accessed: Jun. 24, 2025. [Online]. Available: https://cran.r-project.org/web/packages/dplyr/index.html
[24] K. El Emam, L. Mosquera, and C. Zheng, "Optimizing the synthesis of clinical trial data using sequential trees," *J Am Med Inform Assoc*, Nov. 2020, doi: 10.1093/jamia/ocaa249.
[25] A. Ankan and A. Panda, "pgmpy: Probabilistic Graphical Models using Python," presented at the Python in Science Conference, Austin, Texas, 2015, pp. 6–11. doi: 10.25080/Majora-7b98e3ed-001.
[26] L. Xu, M. Skoularidou, A. Cuesta-Infante, and K. Veeramachaneni, "Modeling Tabular data using Conditional GAN," in *Advances in Neural Information Processing Systems*, vol. 32, 2019. [Online]. Available: https://papers.nips.cc/paper/2019/hash/254ed7d2de3b23ab10936522dd547b78-Abstract.html
[27] D. S. Watson, K. Blesch, J. Kapar, and M. N. Wright, "Adversarial random forests for density estimation and generative modeling," Mar. 13, 2023, *arXiv*: arXiv:2205.09435. doi: 10.48550/arXiv.2205.09435.
[28] C. Durkan, A. Bekasov, I. Murray, and G. Papamakarios, "Neural Spline Flows," Dec. 02, 2019, *arXiv*: arXiv:1906.04032. doi: 10.48550/arXiv.1906.04032.
[29] K. E. Emam, S. E. Kababji, L. Pilgram, V. Cano, and D. Liu, "pysdg," Jul. 2024, doi: 10.17605/OSF.IO/XJ9PR.
[30] T. Hothorn, K. Hornik, and A. Zeileis, "Unbiased Recursive Partitioning: A Conditional Inference Framework," *Journal of Computational and Graphical Statistics*, vol. 15, no. 3, pp. 651–674, Sep. 2006, doi: 10.1198/106186006X133933.
[31] J. Read, B. Pfahringer, G. Holmes, and E. Frank, "Classifier Chains for Multi-label Classification," in *Machine Learning and Knowledge Discovery in Databases*, W. Buntine, M. Grobelnik, D. Mladenić, and J. Shawe-Taylor, Eds., in Lecture Notes in Computer Science. Berlin, Heidelberg: Springer, 2009, pp. 254–269. doi: 10.1007/978-3-642-04174-7_17.
[32] J. Drechsler and J. P. Reiter, "An empirical evaluation of easily implemented, nonparametric methods for generating synthetic datasets," *Computational Statistics & Data Analysis*, vol. 55, no. 12, pp. 3232–3243, Dec. 2011, doi: 10.1016/j.csda.2011.06.006.
[33] R. C. Arslan, K. M. Schilling, T. M. Gerlach, and L. Penke, "Using 26,000 diary entries to show ovulatory changes in sexual desire and behavior," *J Pers Soc Psychol*, vol. 121, no. 2, pp. 410–431, 2021, doi: 10.1037/pspp0000208.
[34] D. Bonnéry *et al.*, "The Promise and Limitations of Synthetic Data as a Strategy to Expand Access to State-Level Multi-Agency Longitudinal Data," *Journal of Research on Educational Effectiveness*, vol. 12, no. 4, pp. 616–647, Oct. 2019, doi: 10.1080/19345747.2019.1631421.
[35] A. Sabay, L. Harris, V. Bejugama, and K. Jaceldo-Siegl, "Overcoming Small Data Limitations in Heart Disease Prediction by Using Surrogate Data," *SMU Data Science Review*, vol. 1, no. 3, p. Article 12, Aug. 2018.
[36] Michael Freiman, Amy Lauger, and Jerome Reiter, "Data Synthesis and Perturbation for the American Community Survey at the U.S. Census Bureau," US Census Bureau. https://www.census.gov/library/working-papers/2018/adrm/formal-privacy-synthetic-data-acs.html, Working paper, 2017. Accessed: Feb. 24, 2020. [Online]. Available: https://www.census.gov/library/working-papers/2018/adrm/formal-privacy-synthetic-data-acs.html
[37] B. Nowok, "Utility of synthetic microdata generated using tree-based methods," presented at the UNECE Statistical Data Confidentiality Work Session, Helsinki, Oct. 2015. doi: https://unece.org/statistics/events/SDC2015.
[38] G. M. Raab, B. Nowok, and C. Dibben, "Practical Data Synthesis for Large Samples," *Journal of Privacy and Confidentiality*, vol. 7, no. 3, pp. 67–97, 2016, doi: 10.29012/jpc.v7i3.407.

[39] B. Nowok, G. M. Raab, and C. Dibben, “Providing bespoke synthetic data for the UK Longitudinal Studies and other sensitive data with the synthpop package for R 1,” *Statistical Journal of the IAOS*, vol. 33, no. 3, pp. 785–796, Jan. 2017, doi: 10.3233/SJI-150153.
[40] D. S. Quintana, “A synthetic dataset primer for the biobehavioural sciences to promote reproducibility and hypothesis generation,” *eLife*, vol. 9, p. e53275, 2020, doi: 10.7554/eLife.53275.
[41] D. Kaur *et al.*, “Application of Bayesian networks to generate synthetic health data,” *J Am Med Inform Assoc*, vol. 28, no. 4, pp. 801–811, Mar. 2021, doi: 10.1093/jamia/ocaa303.
[42] K. P. Murphy, *Machine Learning: A Probabilistic Perspective*. MIT Press, 2012.
[43] Z. Qian, B.-C. Cebere, and M. van der Schaar, “Synthcity: facilitating innovative use cases of synthetic data in different data modalities,” *arXiv*, vol. 2301.07573, Jan. 2023, Accessed: Oct. 10, 2023. [Online]. Available: https://arxiv.org/abs/2301.07573v1
[44] I. Goodfellow *et al.*, “Generative adversarial nets,” in *Advances in neural information processing systems*, 2014, pp. 2672–2680.
[45] S. Bourou, A. El Saer, T. Velivasaki, A. Voulkidis, and T. Zahariadis, “A Review of Tabular Data Synthesis Using GANs on an IDS Dataset,” *Information*, vol. 12, p. 375, Sep. 2021, doi: 10.3390/info12090375.
[46] D. P. Kingma and M. Welling, “Auto-Encoding Variational Bayes,” Dec. 2013. doi: 10.48550/arXiv.1312.6114.
[47] Z. Wan, Y. Zhang, and H. He, “Variational autoencoder based synthetic data generation for imbalanced learning,” in *2017 IEEE Symposium Series on Computational Intelligence (SSCI)*, Nov. 2017, pp. 1–7. doi: 10.1109/SSCI.2017.8285168.
[48] H. Ishfaq, A. Hoogi, and D. Rubin, “TVAE: Triplet-Based Variational Autoencoder using Metric Learning,” Feb. 08, 2023, *arXiv*: arXiv:1802.04403. doi: 10.48550/arXiv.1802.04403.
[49] K. Sohn, H. Lee, and X. Yan, “Learning Structured Output Representation using Deep Conditional Generative Models,” in *Advances in Neural Information Processing Systems*, Curran Associates, Inc., 2015. Accessed: Aug. 14, 2024. [Online]. Available: https://papers.nips.cc/paper/2015/hash/8d55a249e6baa5c06772297520da2051-Abstract.html
[50] A. Salim, *Synthetic Patient Generation: A Deep Learning Approach Using Variational Autoencoders*. 2018.
[51] H. Akrami, A. A. Joshi, J. Li, S. Aydöre, and R. M. Leahy, “A robust variational autoencoder using beta divergence,” *Knowledge-Based Systems*, vol. 238, p. 107886, Feb. 2022, doi: 10.1016/j.knosys.2021.107886.
[52] J. Snoek, H. Larochelle, and R. P. Adams, “Practical Bayesian optimization of machine learning algorithms,” in *Proceedings of the 25th International Conference on Neural Information Processing Systems - Volume 2*, in NIPS’12. Red Hook, NY, USA: Curran Associates Inc., Dec. 2012, pp. 2951–2959. doi: https://papers.nips.cc/paper_files/paper/2012/hash/05311655a15b75fab86956663e1819cd-Abstract.html.
[53] E. Bartz, T. Bartz-Beielstein, M. Zaefferer, and O. Mersmann, Eds., *Hyperparameter Tuning for Machine and Deep Learning with R: A Practical Guide*. Singapore: Springer Nature, 2023. doi: 10.1007/978-981-19-5170-1.
[54] B. Bischl *et al.*, “Hyperparameter Optimization: Foundations, Algorithms, Best Practices and Open Challenges,” arXiv.org. Accessed: Dec. 09, 2023. [Online]. Available: https://arxiv.org/abs/2107.05847v3
[55] M. Binder, F. Pfisterer, and B. Bischl, “Collecting Empirical Data About Hyperparameters for Data Driven AutoML,” *7th ICML Workshop on Automated Machine Learning*, 2020.

[56] D. Kühn, P. Probst, J. Thomas, and B. Bischl, “Automatic Exploration of Machine Learning Experiments on OpenML,” arXiv.org. Accessed: Dec. 09, 2023. [Online]. Available: https://arxiv.org/abs/1806.10961v3

[57] L. Juwara, A. El-Hussuna, and K. El Emam, “An evaluation of synthetic data augmentation for mitigating covariate bias in health data,” *Patterns*, 2024, doi: 10.1016/j.patter.2024.100946.

[58] Y. Huang, W. Li, F. Macheret, R. A. Gabriel, and L. Ohno-Machado, “A tutorial on calibration measurements and calibration models for clinical prediction models,” *J Am Med Inform Assoc*, vol. 27, no. 4, pp. 621–633, Apr. 2020, doi: 10.1093/jamia/ocz228.

[59] M. Kull, T. S. Filho, and P. Flach, “Beta calibration: a well-founded and easily implemented improvement on logistic calibration for binary classifiers,” in *Proceedings of the 20th International Conference on Artificial Intelligence and Statistics*, PMLR, Apr. 2017, pp. 623–631. doi: https://proceedings.mlr.press/v54/kull17a.html.

[60] K. E. Emam, “sdgm Package,” Jan. 2024, doi: 10.17605/OSF.IO/DCJM6.

[61] “TensorFlow for R - Reference.” Accessed: Jan. 22, 2025. [Online]. Available: https://tensorflow.rstudio.com/reference/

[62] A. Kadra, M. Lindauer, F. Hutter, and J. Grabocka, “Well-tuned Simple Nets Excel on Tabular Datasets,” Nov. 05, 2021, *arXiv*: arXiv:2106.11189. doi: 10.48550/arXiv.2106.11189.

[63] J. Salinas Ruíz, O. A. Montesinos López, G. Hernández Ramírez, and J. Crossa Hiriart, “Generalized Linear Mixed Models for Proportions and Percentages,” in *Generalized Linear Mixed Models with Applications in Agriculture and Biology*, Springer, Cham, 2023, pp. 209–278. doi: 10.1007/978-3-031-32800-8_6.

[64] D. Bates, M. Mächler, B. Bolker, and S. Walker, “Fitting Linear Mixed-Effects Models Using lme4,” *Journal of Statistical Software*, vol. 67, pp. 1–48, Oct. 2015, doi: 10.18637/jss.v067.i01.

[65] A. Kuznetsova, P. B. Brockhoff, and R. H. B. Christensen, “lmerTest Package: Tests in Linear Mixed Effects Models,” *Journal of Statistical Software*, vol. 82, pp. 1–26, Dec. 2017, doi: 10.18637/jss.v082.i13.

[66] K. Bartoń, *MuMIn: Multi-Model Inference*. (Apr. 01, 2025). Accessed: May 20, 2025. [Online]. Available: https://cran.r-project.org/web/packages/MuMIn/index.html
